# *SEC24C* deficiency causes trafficking and glycosylation abnormalities in an epileptic encephalopathy with cataracts and dyserythropoeisis

**DOI:** 10.1172/jci.insight.173484

**Published:** 2025-03-25

**Authors:** Nina Bögershausen, Büsranur Cavdarli, Taylor H. Nagai, Miroslav P. Milev, Alexander Wolff, Mahsa Mehranfar, Julia Schmidt, Dharmendra Choudhary, Óscar Gutiérrez-Gutiérrez, Lukas Cyganek, Djenann Saint-Dic, Arne Zibat, Karl Köhrer, Tassilo E. Wollenweber, Dagmar Wieczorek, Janine Altmüller, Tatiana Borodina, Dilek Kaçar, Göknur Haliloğlu, Yun Li, Christian Thiel, Michael Sacher, Ela W. Knapik, Gökhan Yigit, Bernd Wollnik

**Affiliations:** 1 Institute of Human Genetics, University Medical Center Göttingen, Göttingen, Germany.; 2 Department of Medical Genetics, Ankara Bilkent City Hospital, Ankara, Turkey.; 3 Department of Medical Genetics, Ankara Yildirim Beyazit University, Ankara, Turkey.; 4 Vanderbilt Genetics Institute and Vanderbilt University Medical Center, Nashville, Tennessee, USA.; 5 Department of Cell and Developmental Biology, Vanderbilt University, Nashville, Tennessee, USA.; 6 Department of Biology, Concordia University, Montréal, Québec, Canada.; 7 German Center for Child and Adolescent Health (DZKJ), partner site Göttingen, Göttingen, Germany.; 8 Department of Chemistry and Biochemistry, Concordia University, Montréal, Québec, Canada.; 9 Stem Cell Unit, Department of Cardiology and Pneumology, University Medical Center Göttingen, Göttingen, Germany.; 10 Translational Neuroinflammation and Automated Microscopy TNM, Fraunhofer Institute for Translational Medicine and Pharmacology ITMP, Göttingen, Germany.; 11 German Centre for Cardiovascular Research (DZHK), partner site Lower Saxony, Lower Saxony, Germany.; 12 Cluster of Excellence Multiscale Bioimaging: From Molecular Machines to Networks of Excitable Cells (MBExC), University of Göttingen, Göttingen, Germany.; 13 Genomics & Transcriptomics Laboratory, Biological and Medical Research Center (BMFZ), and; 14 Institute of Human Genetics, Medical Faculty and University Hospital Düsseldorf, Heinrich-Heine-University, Düsseldorf, Germany.; 15 Berlin Institute of Health at Charité – Universitätsmedizin Berlin, Berlin, Germany.; 16 Max Delbrück Center for Molecular Medicine, Berlin, Germany.; 17 Department of Pediatric Hematology, Ankara Bilkent City Hospital, Ankara, Turkey.; 18 Division of Pediatric Neurology, Department of Pediatrics, Hacettepe University Faculty of Medicine, Ankara, Turkey.; 19 Center for Pediatric and Adolescent Medicine, Department I, Heidelberg University, Heidelberg, Germany.; 20 Department of Anatomy and Cell Biology, McGill University, Montréal, Québec, Canada.

**Keywords:** Genetics, Neuroscience, Epilepsy, Glycobiology, Protein traffic

## Abstract

As a major component of intracellular trafficking, the coat protein complex II (COPII) is indispensable for cellular function during embryonic development and throughout life. The 4 SEC24 proteins (A–D) are essential COPII components involved in cargo selection and packaging. A human disorder corresponding to alterations of SEC24 function is currently known only for *SEC24D*. Here, we reported that biallelic loss of SEC24C leads to a syndrome characterized by primary microcephaly, brain anomalies, epilepsy, hearing loss, liver dysfunction, anemia, and cataracts in an extended consanguineous family with 4 affected individuals. We showed that knockout of *sec24C* in zebrafish recapitulated important aspects of the human phenotype. SEC24C-deficient fibroblasts displayed alterations in the expression of several COPII components as well as impaired anterograde trafficking to the Golgi, indicating a severe impact on COPII function. Transcriptome analysis revealed that SEC24C deficiency also affected the proteasome and autophagy pathways. Moreover, a shift in the N-glycosylation pattern and deregulation of the N-glycosylation pathway suggested a possible secondary alteration of protein glycosylation, linking the described disorder with the congenital disorders of glycosylation.

## Abstract 2

Deficiency of *SEC24C* causes a syndromic epileptic encephalopathy, alters important cellular pathways and causes lens defects, cerebellar anomalies and locomotor defects in a zebrafish model.

## Graphical abstract

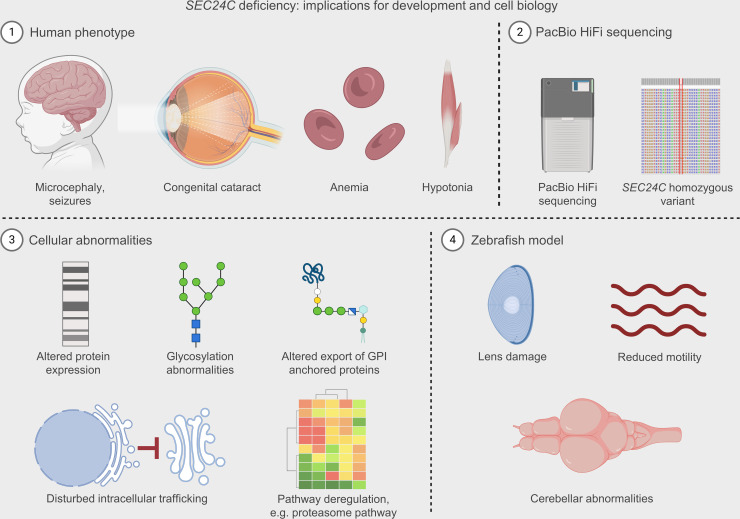

## Introduction

The transport of molecules from the endoplasmic reticulum (ER) to the Golgi apparatus is an indispensable step in the secretory pathway. For exiting the ER, cargo molecules are packaged into vesicular carriers that bud from ER exit sites and then travel to and fuse with the cis-Golgi network. The coat protein complex II (COPII) is a crucial player in ER exit. Among the main COPII components, the 4 SEC24 paralogs (A–D) fulfill specific functions in cargo recognition and selection (1). SEC24C, for example, shows specificity for glycosyl phosphatidylinositol–anchored (GPI-anchored) or IxM motif–containing proteins, such as the soluble *N*-ethylmaleimide–sensitive factor attachment protein receptor proteins GOSR2 and STX5 (1). Cargo selectivity and differential spatiotemporal expression likely underlie the phenotypic outcomes of loss of any of the *Sec24* genes in mice (2). In humans, *SEC24D* is currently the only gene of the *SEC24* family that has been associated with a syndrome. *SEC24D* deficiency leads to an autosomal-recessive syndromic form of osteogenesis imperfecta and autosomal-recessive Cole-Carpenter syndrome 2 (OMIM 616294) (3, 4), due to impaired SEC24D-dependent procollagen export from the ER.

Here, we describe a consanguineous Turkish family with 4 individuals affected by a syndromic disorder characterized by dysmorphic facial features, congenital cataracts, optic atrophy, hearing loss, primary microcephaly and brain anomalies, drug-resistant epileptic encephalopathy, congenital anemia, hepatosplenomegaly, and dyslipidemia. Long-read genome sequencing identified the causative, homozygous frameshift variant c.333del p.(Ser112Profs*115) in the *SEC24C* gene, which led to loss of SEC24C and severely impaired ER-Golgi trafficking in patient-derived fibroblasts. Transcriptome analysis and mass spectrometry revealed an impact on the N-glycosylation machinery as well as a deregulation of the proteasome and autophagy pathways. Moreover, several important phenotypes were recapitulated in a zebrafish CRISPR/Cas9-induced knockout of *sec24C* and a *sec24C/D* double knockout.

## Results

### Clinical report.

**Figure 1:**
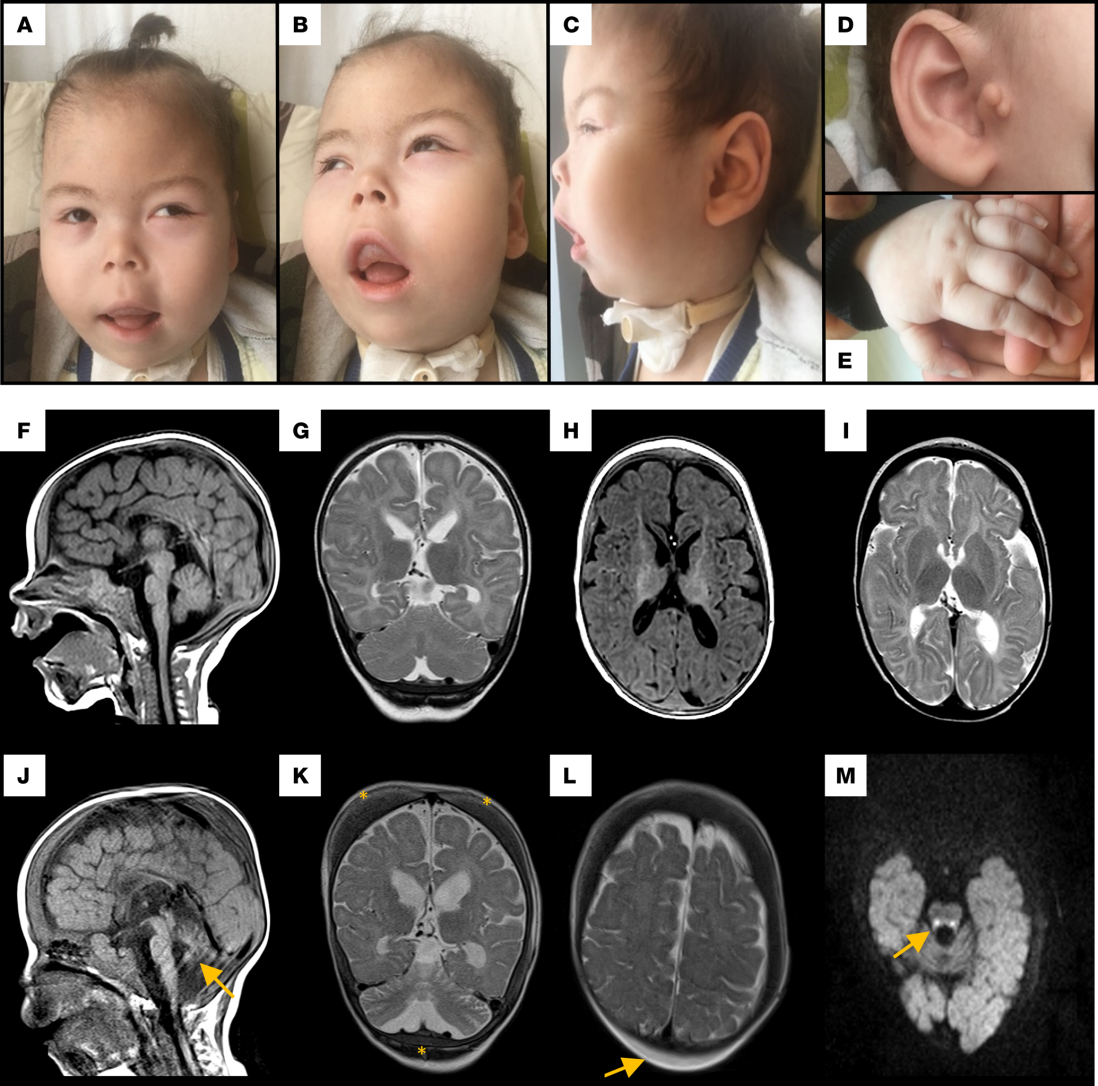
Clinical findings and MRI results of individual V-8. (**A**–**C**) Note full cheeks, small nose with hypoplastic alae, hypoplastic malar region, long philtrum, arched and thin upper lip, gingival hypertrophy, right preauricular tags (**D**), and tapering fingers (**E**). (**F**–**I**) Cranial MRI at the age of 7 months and 14 months (**J**–**M**). T1-weighted sagittal (**F** and **J**) and T2-weighted coronal images (**G** and **K**) showing thin corpus callosum, progressive cerebellar atrophy (yellow arrow), cerebral atrophy with enlarged lateral ventricles, and enlargement of calvarial bones demonstrated by increased bone marrow intensity (yellow asterisks). T1- and T2-weighted transverse images with diffuse hypomyelination pattern (**H** and **I**). T2-weighted transverse image shows lack of myelination and increased bilateral parietal subcutaneous fat (yellow arrow) (**L**). Transverse diffusion weighted image b = 1,000 (**M**) demonstrates restricted diffusion in dorsal brainstem pathways at the level of the superior cerebellar peduncle (yellow arrow).

Individual V-8 (Figure 1, A–E) was born after an uneventful pregnancy at 38+6 weeks of gestation, with a birth weight of 2,650 g (–1.53 SDs) and length of 46 cm (–2.05 SDs) (head circumference was not recorded). Birth was complicated by meconium aspiration, and he was admitted to the neonatal intensive care unit (NICU) with acute respiratory distress. He required respiratory support for a day and a nasogastric tube for feeding due to feeding and swallowing problems. During his stay at the NICU, he was noticed to have severe cerebral hypotonia, and he received surgery because of bilateral congenital cataracts. He had neonatal onset seizures treated with phenobarbital and levetiracetam. After being discharged from the NICU, at the age of 2 months, he had a focal onset generalized tonic seizure lasting for 5 minutes leading to respiratory difficulties and a respiratory arrest upon admission to the emergency department. He had been treated with different combinations of antiseizure medications and received a gastrostomy tube due to swallowing dysfunction and a tracheostomy due to respiratory insufficiency by the age of 7 months. Neonatal onset seizures evolved to bilateral frontoparietal epileptic discharges at 3 months of age, burst suppression pattern at 4 months of age, and multifocal epilepsy at 5 months of age. At his most recent follow-up visit, at 4 10/12 years of age, he had ongoing daily seizures during arousal or sleep, lasting for about 10–15 seconds and characterized by short episodes of apnea, abnormal laugh, eye blinking, and right lower extremity clonic movements. He also had tonic generalized seizures with a variable frequency and is currently on a combination therapy. Developmental milestones were severely delayed. He never achieved head control and was never able to sit even with support. He did not recognize his mother and had no verbal output. Past medical history revealed gallbladder stone, hypopotassemia, recurrent urinary tract infections, and a patent foramen ovale. He also had marked gingival hyperplasia (Figure 1B) and no primary dentition. However, one of his healthy sisters also has, though much milder, gingival hyperplasia and oligodontia.

Head circumference was 39 cm (–2.82 SDs), 40 cm (–3.76 SDs), and 45 cm (–2.31 SDs) by the ages of 5 months, 8 months, and 20 months, respectively. By 3 years and 4 10/12 years, his head circumference had increased to 51 cm (0.83 SDs) and 51.5 cm (–0.02 SDs), respectively. An increase in parietal subcutaneous fatty tissue and an enlargement of the calvaria, head base, and facial bones correlated with the change in head circumference. His weight was 17 kg (–0.55 SDs) and his height was 105 cm (–0.97 SDs) at age 4 10/12 years.

Splenomegaly was appreciated on physical examination. Dysmorphic facial features included full cheeks; a very small, sunken nose with hypoplastic alae and flared nostrils; a hypoplastic malar region; a long philtrum; a high-arched upper lip (Figure 1, A–C); gingival hyperplasia, and preauricular tags on the right side (Figure 1D). He also had tapering fingers (Figure 1E). He was dependent on mechanical ventilation through a tracheostomy and feeding through a gastrostomy tube. He was unresponsive to his environment, did not follow objects, had no eye contact, and had severe developmental delay and severe intellectual disability. He showed no response to voices or objects and had anisocoria, nystagmoid eye movements, bilateral optic atrophy, and tongue fasciculations. Furthermore, he showed peripheral hypotonia, absence of deep tendon reflexes, head lag on traction posture, and no voluntary movements against gravity in all extremities, accompanied by mild knee flexion contractures.

Review of the laboratory workup revealed congenital macrocytic anemia, leukopenia, and thrombocytopenia, and a blood smear at 2 years and 8 months of age showed anisocytosis, spherocytosis, and sphero-acanthocytes as well as enlarged platelets (Supplemental Figure 1; supplemental material available online with this article; https://doi.org/10.1172/jci.insight.173484DS1). A bone marrow biopsy was performed in the first year of life to rule out hemophagocytic lymphohistiocytosis. Brainstem evoked response audiometry showed profound bilateral sensorineural hearing loss. Abdominal ultrasonography demonstrated hepatosplenomegaly and enlarged surrenal glands. Liver enzymes were continuously elevated as well as ferritin, triglycerides, and alpha-fetoprotein (Supplemental Table 1). Cranial MRI at the age of 7 months revealed thin corpus callosum and diffuse hypomyelination (Figure 1, F–I). A repeat MRI study at the age of 14 months showed progressive cerebellar, predominantly vermian, and brainstem atrophy as well as cerebral atrophy with dilated lateral ventricles; diffuse hypomyelination; enlarged calvarial, head base, and facial bones; restricted diffusion on dorsal brainstem pathways; and increase in subcutaneous fatty tissue in the parietal region bilaterally (Figure 1, J–M). The MRI also revealed bilateral, postsurgery absence of the lens and optic nerve atrophy (images not shown). Recent awake electroencephalography revealed diffuse abnormalities on background activity characterized by almost continuous rhythmic spike wave discharges in the temporal region bilaterally, as well as bilateral parasagittal suppression compatible with an epileptic encephalopathy. The patient passed away at the age of 5 years, due to a respiratory infection.

### Family history.

Individual V-8 was the third child of a healthy consanguineous Turkish couple. He has 2 healthy sisters. His mother experienced intrauterine fetal demise in the sixth month of another pregnancy. The cause of death of the fetus is uncertain. In addition, individual V-8 had 3 third cousins who most likely were affected by the same disorder. These deceased cousins were reported to experience epilepsy, cataracts, and intellectual disability. The individual’s parents are first cousins, the affected cousins’ parents are first cousins once removed, and there is a high rate of consanguinity in the family. Unfortunately, there was no biological material of the deceased cousins remaining for investigation. However, we were able to collect blood samples from the cousins’ parents and their healthy sister, as well as 4 healthy members from another branch of the family.

### Identification of a causative, homozygous SEC24C variant.

To identify the genetic cause of the presented phenotype, we performed exome sequencing (ES) on DNA of individual V-8, followed by PacBio long-read genome sequencing (lrWGS). The long-read dataset achieved an average percentage identity of 98.7%. The mean read length was approximately 15,600 bases, with an N50 of around 17,000 bases. A total of 6 million reads were generated, and the median read quality was approximately 32, indicating high data accuracy.

**Figure 2:**
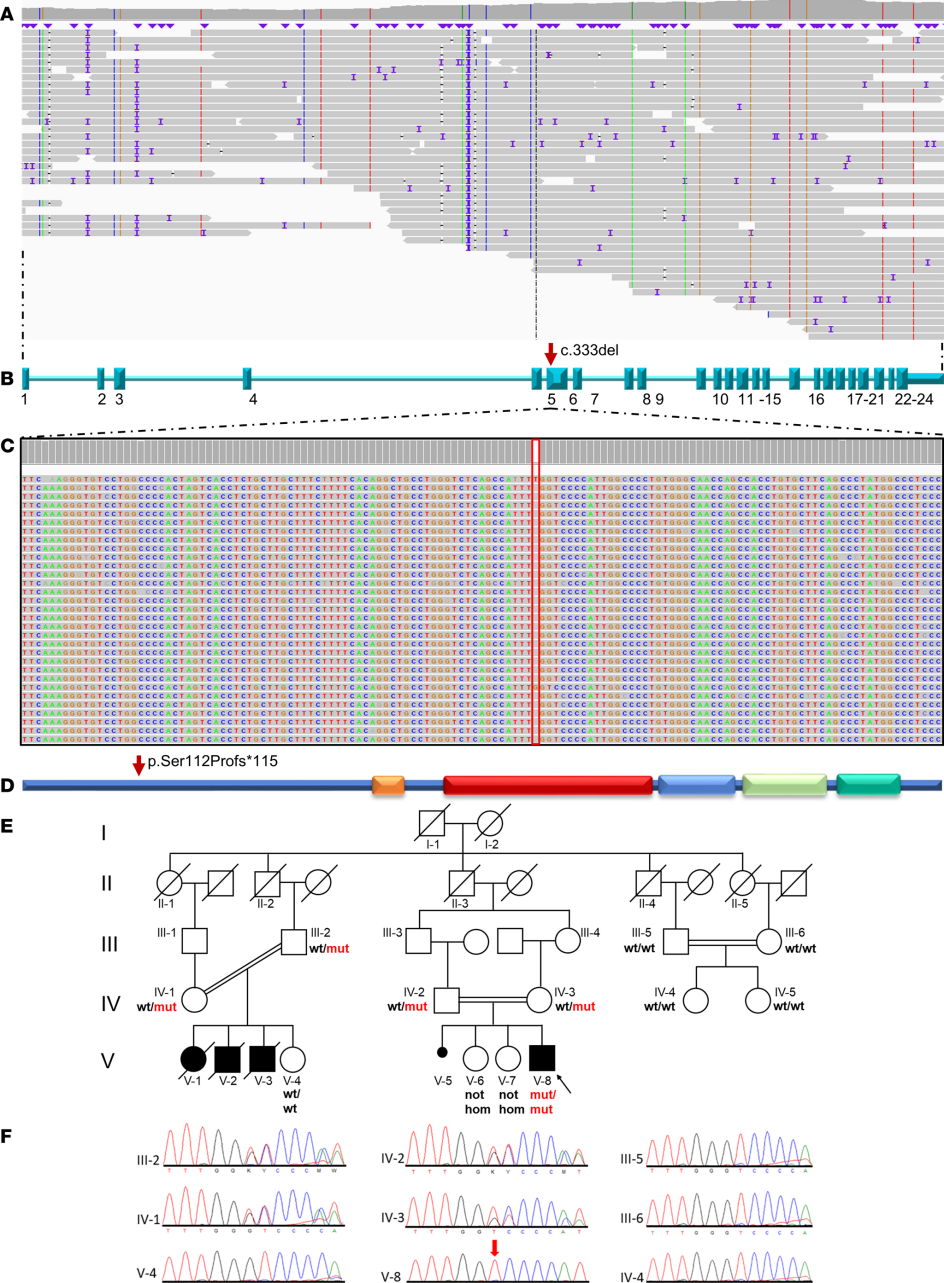
Long-read genome sequencing shows the homozygous c.333del variant. (**A**) Long reads show robust coverage of *SEC24C*. (**B**) Schematic representation of the *SEC24C* gene. Exons are depicted as blue rectangles and consecutively numbered. (**C**) Zoom into exon 5 of *SEC24C* showing the homozygous variant c.333del. (**D**) Schematic representation of the SEC24C protein. Domains are indicated as colored boxes. Orange: zinc finger domain; red: trunk domain; blue: β-sandwich domain; light green: helical domain; turquoise: gelsolin-like domain. Amino acid positions according to pfam are indicated below the schematic. The variant is indicated by a red arrow in the gene and protein representations. (**E**) Five-generation pedigree showing consanguineous marriages and cosegregation of the c.333del variant. hom., homozygous for the c.333del variant; wt, wild-type; mut, mutation. (**F**) Electropherograms showing the *SEC24C* c.333del variant (red arrow) in individual V-8 (homozygous), his parents (heterozygous), and the affected cousins’ parents (heterozygous). The electropherograms of V-6 and V-7 are not shown, for ethical reasons.

In view of the parental consanguinity, we focused our filtering strategy on rare homozygous variants with an impact predicted as high or moderate, and we identified the homozygous variant NM_004922.3:c.333del (NC_000010.11:g.73759646del) in the *SEC24C* gene (OMIM 607185) (Figure 2, A–D). Using lrWGS, no additional likely causative biallelic single nucleotide variants (SNVs) or structural variants (SVs) were identified in noncoding parts of the genome (Supplemental Data 1 and 2). AI-based prioritization of SNVs within the lrWGS dataset using the Human Phenotype Ontology (HPO) terms “anemia,” “microcephaly,” “intellectual disability,” “hearing impairment,” “cataract,” and “abnormality of the liver” supported the homozygous variant c.333del in *SEC24C* as the most likely causative biallelic variant.

To exclude important differential diagnoses, we searched the ES and lrWGS data for variants in genes associated with hyperferritinemia-cataract syndrome (OMIM 600886), transient neonatal hypertriglyceridemia (OMIM 138420), dyslipoproteinemic corneal dystrophy (OMIM 136120), and mevalonic aciduria (OMIM 610377): *FTL*, *GPD1*, *LCAT*, and *MVK*, respectively. We also excluded variants in genes associated with Fanconi anemia and other phenotypes characterized by a combination of developmental abnormalities, microcephaly, and anemia as well as genes associated with congenital cataracts, without finding a pathogenic variant. An HPO-based analysis using the terms gingival overgrowth (HP:0000212) and oligodontia (HP:0000677) revealed no additional likely causative variants.

Both unaffected parents are heterozygous carriers of the *SEC24C* c.333del variant (Figure 2, E and F). The healthy sisters do not carry the c.333del variant in the homozygous state (electropherograms not shown because the individuals are underage). The unaffected parents of the affected cousins (individuals IV-1 and III-2) are heterozygous carriers of the *SEC24C* c.333del variant, whereas their unaffected daughter (V-4) is not a carrier (Figure 2, E and F). The unaffected individuals III-5, III-6, IV-4, and IV-5 also do not carry the *SEC24C* c.333del variant (Figure 2, E and F).

### Quantitative PCR shows near-complete absence of SEC24C mRNA.

The deletion of a G at position 333 is predicted to lead to a frameshift and a premature termination of protein translation at position 226: p.(Ser112Profs*115) (Figure 2D). The variant c.333del is absent from the Single Nucleotide Polymorphism Database (dbSNP) and the Genome Aggregation Database (gnomAD). Due to the position of this variant in the N-terminal part of the protein, we hypothesized that it would lead to nonsense-mediated decay (NMD) and thus to complete loss of the SEC24C protein.

To test this hypothesis, we performed quantitative PCR (qPCR) on RNA extracted from cultured fibroblasts of individual V-8. We observed a marked reduction of *SEC24C* mRNA by approximately 90% in patient fibroblasts (Supplemental Figure 2). This result indicates that mutant *SEC24C* mRNA is unstable and likely subject to NMD.

### N-glycan analysis reveals truncated sugar structures in serum from individual V-8.

**Figure 3:**
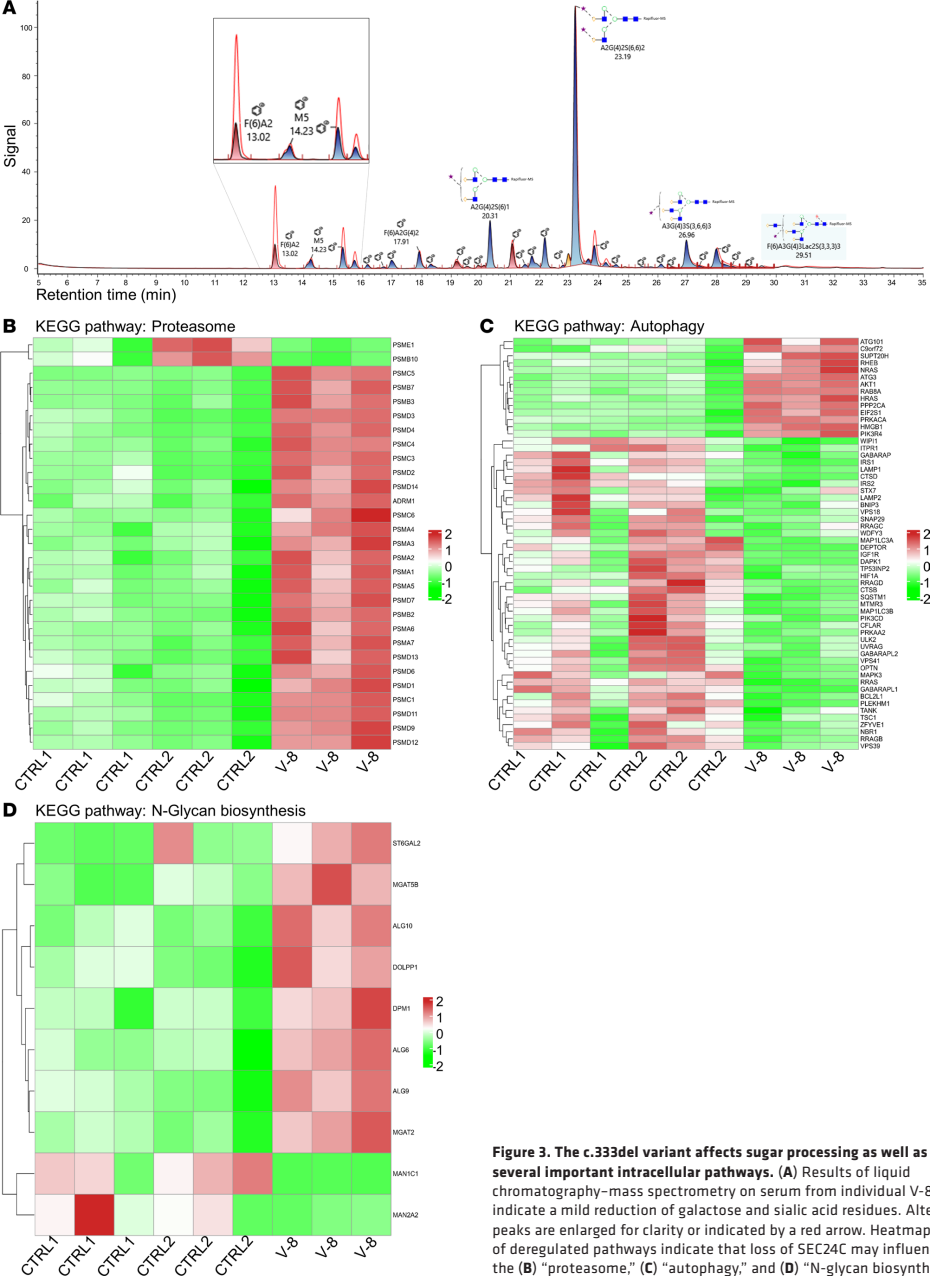
The c.333del variant affects sugar processing as well as several important intracellular pathways. (**A**) Results of liquid chromatography–mass spectrometry on serum from individual V-8 indicate a mild reduction of galactose and sialic acid residues. Altered peaks are enlarged for clarity or indicated by a red arrow. Heatmaps of deregulated pathways indicate that loss of SEC24C may influence the (**B**) “proteasome,” (**C**) “autophagy,” and (**D**) “N-glycan biosynthesis” pathways (*n* = 3/group).

N-glycan analysis of whole serum glycoproteins by liquid chromatography–mass spectrometry (LC-MS) revealed a normal distribution of N-glycans in the patient sample with regard to complex type, hybrid type, and mannose-rich type. Regarding the microheterogeneity of N-glycans, however, a number of truncated sugar structures were present at elevated levels in the sample of individual V-8. Particularly, F(6)A2 (+172.9%), F(6)A2G1 (+52.1%), and F(6)A2G1S1 (+170.0%) showed a strong increase in comparison with the control pool (Figure 3A), indicating reduced transfer of galactose and sialic acid residues by Golgi glycosyltransferases.

### Transcriptome analysis reveals alterations in proteasome, autophagy, and N-glycan biosynthesis pathways.

We performed transcriptome analysis to gain insights into SEC24C deficiency–associated pathway alterations. Bulk RNA sequencing generated over 150 million reads per sample, achieving an average unique mapping length of approximately 206 bp with a mapping rate of 91.01%. Quality metrics consistently indicated high read quality, with nucleotide base Phred scores of minimum 39 (range 0–40).

As expected, and in line with the qPCR results, we observed significantly reduced expression of *SEC24C* with a logarithmic fold-change (logFC) of –2.022 and an adjusted *P* value of 0.00000402. *SEC24D* expression was slightly elevated with a logFC of 0.2575 (adjusted *P* value 0.2724). RNA levels of *SEC24A* and *SEC24B* were unchanged compared with controls.

Overrepresentation analysis showed 12 significantly deregulated Kyoto Encyclopedia of Genes and Genomes (KEGG) pathways (Supplemental Data 3). Among these pathways was the proteasome degradation pathway (KEGG pathway hsa03050). Except for the proteasome activator subunit (*PSME1*) and the hematopoiesis-associated subunit *PSMB10*, which were downregulated, a substantial number of proteasome pathway components were significantly upregulated (Figure 3B). Targeted analysis of the KEGG pathway hsa04141, protein processing in the endoplasmic reticulum, showed no significant differences, though several components of this pathway, including components involved in ER-associated degradation, the ubiquitin ligase complex, and proteasome dislocation, were significantly deregulated (data not shown). Activation of proteasomal transport and degradation may indicate a compensatory mechanism, triggered by stalling of SEC24C-specific protein transport. Given these results, we also analyzed the expression of markers of ER stress and the unfolded protein response, such as *IRE1*, *PERK*, *ATF4*, *ATF6*, and *XBP1*, but observed no significant upregulation of these transcripts.

Considering the noncanonical function of SEC24C in autophagy (5), we investigated the autophagy pathway (hsa04140) and observed a significant deregulation of a number of important components (Figure 3C). We noticed a downregulation of components involved in autophagosome formation, especially *MAP1LC3A* (LC3). These genes are linked to the lysosome pathway (hsa04142), which was also deregulated with a *P* value of 0.005854628 and an adjusted *P* value of 0.123313106.

Next, we performed a targeted pathway analysis of the N-glycan biosynthesis pathway and observed a deregulation of several components of this pathway (Figure 3D), which was in line with the results from the mass spectrometry N-glycan analysis in patient fibroblasts.

### Immunostaining reveals normal Golgi morphology in V-8 fibroblasts.

**Figure 4:**
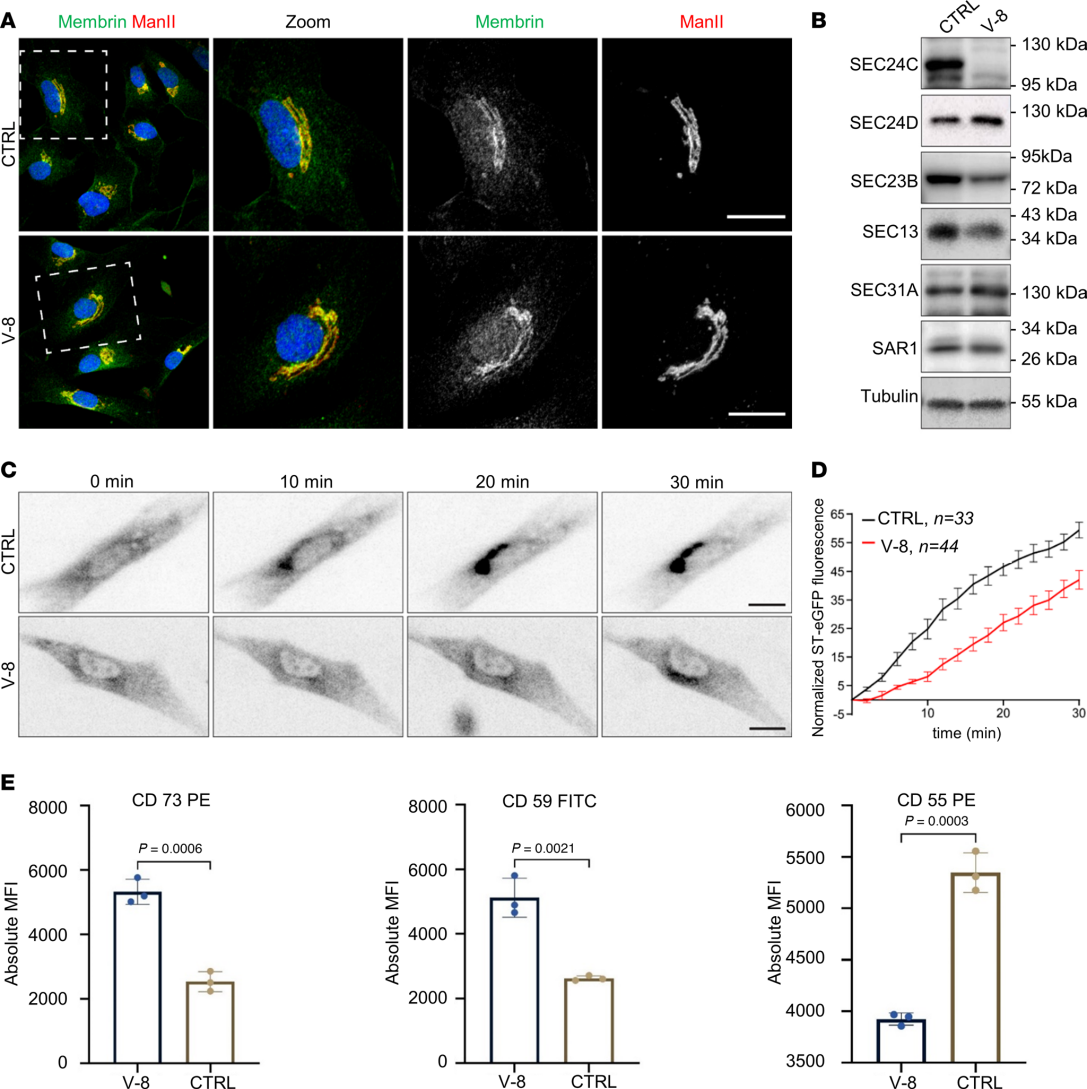
The c.333del p.(Ser112Profs*115) variant affects protein levels of COPII components and intracellular trafficking. (**A**) The Golgi morphology and the localization of GOSR2 (membrin) are not altered in fibroblasts from individual V-8. Representative confocal images of fibroblasts fixed and stained with antibodies recognizing the endogenous membrin/GOSR2 (green) and the Golgi marker ManII (red). Scale bars represent 25 μm. (**B**) Representative immunoblot analysis of lysates prepared from control (CTRL) and V-8 fibroblasts and probed for SEC24C, SEC23B, SEC13, SEC31A, SAR1, and tubulin (as a loading control). Molecular size standards are shown to the right of the blots. *N* = 3. (**C**) The RUSH assay was performed to assess the traffic between the ER and the Golgi. Representative images from the movies used for the quantification are shown at the indicated time points. Scale bars represent 25 μm. (**D**) Cells were imaged every 2 minutes after the addition of biotin to release the cargo molecule ST-eGFP from the ER and quantified for control (CTRL) and V-8 fibroblasts. Graph shows normalized ST-eGFP fluorescence in Golgi region (arbitrary units). (**E**) Surface expression of the GPI-anchored proteins CD55, CD59, and CD73 was assessed by flow cytometry using antibodies specific to each protein and correcting for the background using isotype-specific antibodies. The absolute mean fluorescence intensity (MFI) of samples is shown ± SD, in triplicates. Student’s *t* test was performed to show the data significance level.

Since we observed aberrant N-glycosylation, we investigated the morphology of the Golgi membrane by staining for N-glycan synthesis–associated enzyme mannosidase II (ManII) and SEC24C-dependent cargo GOSR2 (membrin) (1). We observed normal Golgi membrane structure and normal localization of both proteins in fibroblasts from individual V-8 (Figure 4A).

### The p.(Ser112Profs*115) variant results in aberrant levels of COPII components.

As an initial step in addressing the functional consequences of the p.(Ser112Profs*115) variant, we examined the protein expression of other COPII components. We showed by Western blot that full-length SEC24C was not detected in fibroblasts from individual V-8 (Figure 4B). We then examined the levels of the other COPII proteins, including SEC23B, the binding partner of SEC24C in the inner layer of the coat, as well as the outer layer components SEC13 and SEC31. Both SEC23B and SEC13 showed slightly decreased protein levels in V-8 fibroblasts compared with control (Figure 4B). Neither SEC31 nor the GTPase SAR1, which recruits the COPII coat, were affected. Consistent with transcriptome data, SEC24D levels were slightly increased in patient fibroblasts, which might indicate compensatory upregulation (Figure 4B). We concluded that the absence of SEC24C affects the expression of several other COPII proteins and thus impacts ER export.

### The p.(Ser112Profs*115) variant affects transport from the ER to the Golgi.

Given the absence of SEC24C and the aberrant levels of several COPII proteins, we tested whether transport from the ER to the Golgi was affected in V-8 fibroblasts. We employed the Retention Using Selective Hooks (RUSH) assay, in which a GFP-tagged Golgi enzyme (sialyl transferase) is fused to a streptavidin-binding protein (SBP) and retained in the ER by interaction with an ER-localized streptavidin protein. Release from the ER is initiated by biotin addition, and the fluorescent signal arriving in the Golgi is monitored and quantified over time. We observed a marked delay in the arrival of the cargo protein in the Golgi (Figure 4, C and D), consistent with a defect in release of the protein. This is likely a consequence of the defect in COPII coat assembly.

### Loss of SEC24C affects cell surface abundance of GPI-anchored proteins.

A previous study demonstrated that both SEC24C and SEC24D convey the movement of the GPI-anchored protein CD59 to the cell surface (6). Given that the c.333del variant in V-8 affected the expression of both of these proteins, we examined the movement of 3 GPI-anchored proteins, including CD59. The surface levels of 2 proteins, CD59 and CD73, were increased in the patient cells (Figure 4E). This could be explained by the slight increase in SEC24D levels in the V-8 fibroblasts. In contrast, the levels of CD55 were significantly reduced in the patient cells, suggesting that this protein may be exclusively dependent upon SEC24C for ER exit (Figure 4E).

### Brefeldin A treatment indicates mildly delayed Golgi disassembly.

In order to further examine the effects of the *SEC24C* c.333del allele, we examined the disassembly and reassembly of the Golgi during and following treatment with brefeldin A (BFA). This fungal protein causes disassembly of the Golgi in human fibroblasts after short treatments and redistribution of the Golgi into the ER with prolonged treatment (7). When treated for 15 minutes, V-8 fibroblasts and control cells both resulted in Golgi fragmentation. Though the rates were largely the same, we noted that complete Golgi fragmentation was slightly faster at later time points in control fibroblasts compared with those derived from V-8 (Supplemental Figure 3). Upon washout of BFA, the rate of Golgi reassembly was identical between V-8 and control fibroblasts (Supplemental Figure 3).

### Zebrafish sec24C CRISPR-edited larvae present with cataracts, locomotor deficits, and smaller cerebellum.

**Figure 5:**
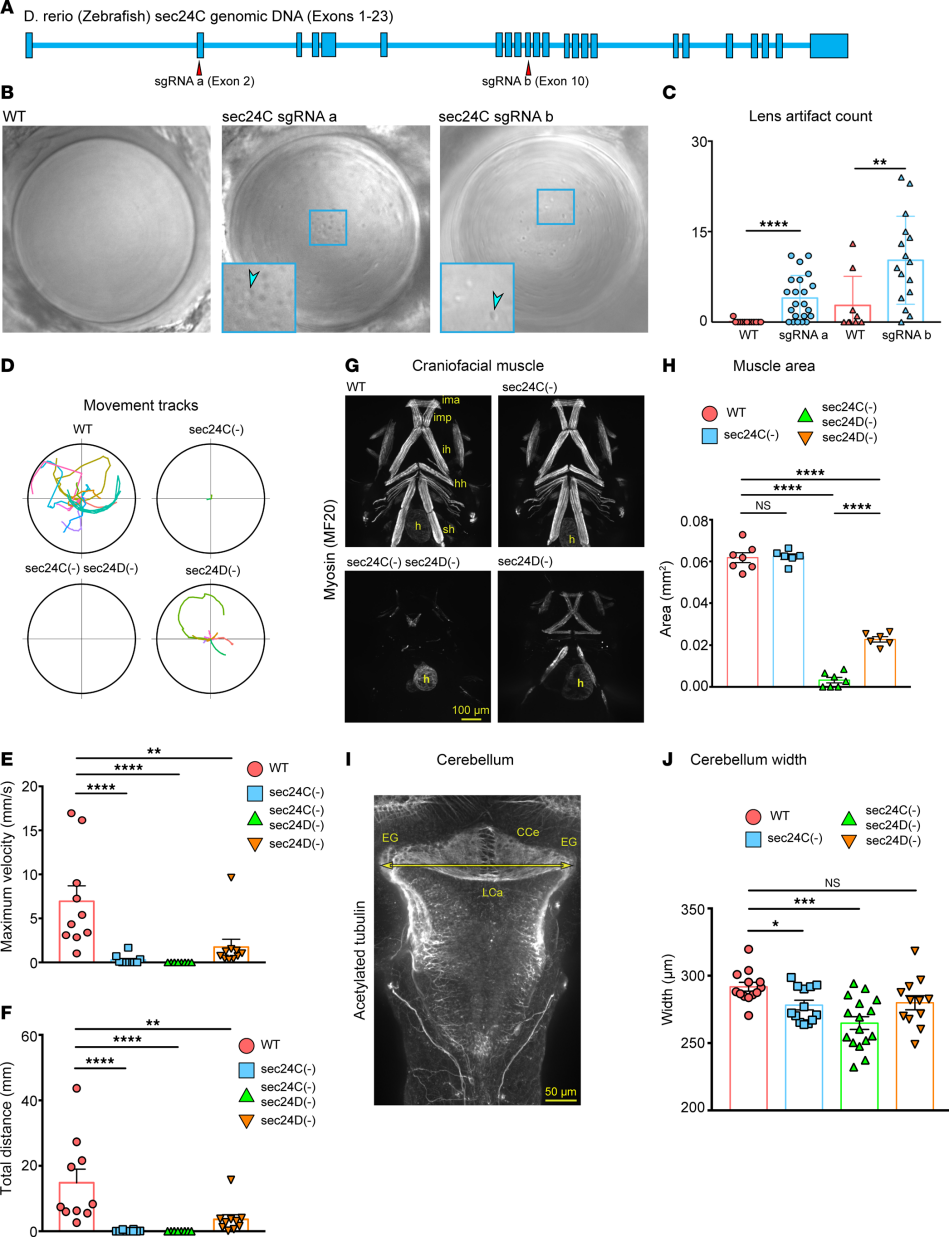
*Sec24C* CRISPR-edited zebrafish larvae present with lens damage, motility impairment, and smaller cerebellum. (**A**) Zebrafish *sec24C* gene overview and localization of single guide RNAs a and b (sgRNA a and sgRNA b), targeting exons 2 and 10, respectively. (**B**) In situ images of lenses in wild-type, sgRNA a *sec24C*(–), and sgRNA b *sec24C*(–) zebrafish larvae at 4 dpf showing lens artifacts. Arrowheads point to droplet-like crystal inclusions in insets. Deep ridges are present along lens circumference in CRISPR-edited lenses. (**C**) Quantification of lens artifacts in single eye per analyzed animal. Graph represents number of artifacts identified per lens/animal sorted by WT (*n* = 13), sgRNA a (*n* = 22), WT (*n* = 9), and sgRNA b (*n* = 16). (**D**) Movement tracks of WT, *sec24C*(–), *sec24C*(–) *sec24D*(–), and *sec24D*(–) zebrafish larvae at 5 dpf. Each colored line represents an individual animal track (*n* = 10 per group). (**E**) Quantification of maximum velocity reached during recorded travel, and (**F**) total distance traveled by each larva. (**G**) Whole-mount immunostaining of craniofacial muscles at 5 dpf in WT, *sec24C*(–), *sec24C*(–) *sec24D*(–), and *sec24D*(–). Scale bar: 100 μm. ima, intermandibularis anterior; imp, intermandibularis posterior; ih, interhyoideus; hh, hyohyoideus; sh, sternohyoideus; h, heart muscle. (**H**) Quantification of craniofacial muscles area in WT (*n* = 7), *sec24C*(–) (*n* = 6), *sec24C*(–) *sec24D*(–) (*n* = 7), and *sec24D*(–) (*n* = 6). (**I**) Whole-mount immunostaining (5 dpf) for acetylated tubulin (axonal tracks), maximum-intensity projection confocal image of a wild-type cerebellum. Double-headed arrow (yellow) marks cerebellum width. Scale bar: 50 μm. EG, eminentia granularis; LCa, lobus caudalis cerebelli; CCe, corpus cerebelli. (**J**) Graph of cerebellum width in WT (*n* = 13), *sec24C*(–) (*n* = 13), *sec24C*(–) *sec24D*(–) (*n* = 16), and *sec24D*(–) (*n* = 12). Statistics: Graphs show mean ± SEM. (**C**, **H**, and **J**) Student’s *t* test. (**E** and **F**) Mann-Whitney *U* test. Significance thresholds: *P < 0.05 or Bonferroni-corrected threshold (**E**, **F**, **H**, and **J**; see Methods), **P < 0.01, ***P < 0.001, ****P < 0.001.

We established 2 independent, transient *sec24C*-knockout zebrafish models using a CRISPR/Cas9 genome-editing strategy. We targeted exon 2 and exon 10 of the zebrafish *sec24C* using single guide RNAs, injected into 1-cell–stage embryos (sgRNA a, sgRNA b, respectively; Figure 5A). We assessed editing efficiency by heteroduplex assays (PCR-based amplification of the targeted region) and direct Sanger sequencing of injected animals (Supplemental Figure 4). We observed 100% editing efficiency in sequenced larvae with most changes resulting in small deletions predicted to generate frameshifts and ultimately stop codons.

Congenital cataracts represent a specific symptom in the proband. Therefore, we examined lenses in live zebrafish at 4 days postfertilization (dpf) and scored morphological differences. Wild-type lenses were transparent, clear, with homogenous smooth features, and concentric layers representing immature fiber cells at the periphery (Figure 5B). On the contrary, *sec24C*-deficient lenses showed deep concentric rings at the periphery of the lens and round, shiny, crystal-like droplets accumulating in the center and sparsely spreading outward across the lens. Both sgRNAs generated similar phenotypes (Figure 5, B and C). Identified artifacts could lead to lens opacity and changes in light scattering, consistent with the cataract phenotype.

The lens data indicated that sec24C is essential for cargos required in lens maturation. However, previous studies (8–10) had shown that the 2 evolutionarily close paralogs, sec24C and sec24D, may have redundant functions during development, and thus, they may compensate for each other’s deficiency. We selected transient knockout models instead of germline-transmitted mutations to avoid phenotypic distortion due to a genetic compensation effect (11, 12). To assess potential functional redundancy, we evaluated phenotypes of single *sec24C*(–) and *sec24D*(–) models and the combined, double *sec24C*(–) *sec24D*(–) knockouts compared with wild-type siblings.

To examine whether the muscular hypotonia in the affected individual is present in the zebrafish model, we tested the swimming behavior. While wild-type siblings responded to a single needle poke and swam away, the CRISPR-edited *sec24C-*deficient larvae were largely unresponsive (Figure 5D). By comparison, *sec24D-*deficient larvae were able to mount a limited response and occasionally swam away. Consistently, double-deficient *sec24C*(–) *sec24D*(–) larvae were unable to respond to the stimulus and did not move. Quantification of maximum velocity of the swim (Figure 5E) and total distance traveled over 30 seconds after the poke (Figure 5F) corroborated observed responses.

Locomotor deficits could stem from musculoskeletal malformations. To test this possibility, we performed whole-mount immunofluorescence staining of muscle myosin (MF20 antibody) and found that craniofacial muscles in *sec24C*(–) zebrafish were normal (Figure 5G), whereas *sec24D*(–) zebrafish showed a decreased craniofacial muscle mass, with the double *sec24C*(–) *sec24D*(–) deficiency having an almost complete loss of craniofacial muscle mass (Figure 5, G and H). It is well accepted that craniofacial muscle induction, differentiation, and maintenance require presence of the neural crest primordia and craniofacial cartilage (13). The double mutant revealed functional redundancy in craniofacial cartilage and muscle development, while there was no effect on the heart muscle expression of muscle myosin (Figure 5G). These results are consistent with the expression of *sec24D* in migrating neural crest primordia and craniofacial cartilage (8), with *sec24C* being expressed at low levels in these tissues. Trunk muscle patterning and expression of muscle myosin were not affected (Supplemental Figure 4D), consistent with no detectable expression of *sec23C* by mRNA staining. Thus, the observed loss of mobility in *sec24C*(–) zebrafish is not caused by muscle loss.

Moreover, the proband was diagnosed with neurological deficits and progressive cerebellar atrophy on MRI examination. Cerebellum in fish is a highly evolutionarily conserved structure where sensory and motor tracks transit through. We performed whole-mount antibody staining against acetylated tubulin to mark axonal tracks that outline the overall brain morphology (Figure 5I and Supplemental Figure 4F). We collected images of the 4 experimental groups at 5 dpf (Figure 5J) and found no major differences between *sec24C*(–) zebrafish and wild-type siblings. The exception was the width of the cerebellum as measured between the left and right EG (Figure 5, I and J). Analyses of zebrafish larvae staining with wheat germ agglutinin (WGA) for N-glycosylated matrix proteins and antibodies recognizing postmitotic neurons (HuC/D) did not reveal significant differences (data not shown). To exclude the possibility that the smaller cerebellum is a consequence of a smaller head or smaller brain, we measured head and brain indices (length-to-width ratio) and did not find significant differences between wild-type siblings and experimental groups (Supplemental Figure 4, E–H), suggesting specific cerebellar deficits.

## Discussion

In this study, we present an extended family in which 4 individuals were affected by a combination of congenital microcephaly and cataracts, severe developmental delay and intellectual disability, and epilepsy. Three of these individuals (V-1, V-2, V-3) were deceased prior to this study. Therefore, only individual V-8 (Figure 1, A–E) was phenotypically described in detail, who also showed congenital anemia, muscular hypotonia, bilateral sensorineural hearing loss, hepatosplenomegaly with elevated liver enzymes, and dyslipidemia. The anemia was characterized as macrocytic anemia with anisocytosis, spherocytosis, and sphero-acanthocytes (Supplemental Figure 1), which, in the absence of hyperbilirubinemia, hints at impaired erythropoiesis. Neonatal onset seizures evolving to epileptic encephalopathy and drug-resistant epilepsy as well as abnormal MRI features, including progressive cerebellar, predominantly vermian, and brainstem atrophy; diffuse hypomyelination; and involvement of dorsal brainstem pathways, were remarkable imaging findings during a follow-up interval of almost 7 months between 2 imaging studies (Figure 1, F–M). Additional striking features in individual V-8 were an increase in parietal subcutaneous fatty tissue; enlargement of the calvaria, the head base, and facial bones; and decreased bone marrow intensity of cranial diploe due to congenital anemia. To the best of our knowledge, this combination of symptoms has not yet been described and constitutes a novel syndrome.

Using a combination of ES and lrWGS, we identified the homozygous truncating variant c.333del p.(Ser112Profs*115) in individual V-8. Since both parents (IV-1 and III-2) of the affected cousins (V-1, V-2, V-3) are heterozygous carriers of the c.333del variant (Figure 2F) and the clinical presentation was very similar, it is highly likely that individuals V-1, V-2, and V-3 had the same *SEC24C*-associated disorder as individual V-8. The variant leads to an early premature stop codon (Figure 2D), resulting in NMD, as demonstrated by severely reduced *SEC24C* mRNA levels (Supplemental Figure 2) and absence of detectable SEC24C protein in V-8 fibroblasts (Figure 4B). lrWGS did not reveal any additional pathogenic or likely pathogenic homozygous noncoding variants that might have caused the phenotype (Supplemental Data 1 and 2).

COPII proteins facilitate the anterograde trafficking of proteins, some of which are glycosylated, from the ER to the Golgi. Several of these glycoproteins belong to the N-glycosylation pathway and are themselves involved in the allocation of sugar moieties to other proteins (14). Thus, we asked whether the loss of SEC24C would affect sugar processing. Notably, we observed a clinical overlap between the phenotype in individual V-8 and the clinical spectrum of congenital disorders of glycosylation, such as microcephaly, epilepsy, congenital cataracts, intellectual disability, hepatosplenomegaly, dyslipidemia, and dysmorphic facial features (15). Analysis of N-glycans of whole serum glycoproteins by LC-MS revealed some sugar moieties that were shortened by the absence of galactose and sialic acids, indicating at least a mild impact on protein glycosylation as a consequence of the loss of SEC24C or as a secondary effect of disturbed anterograde trafficking (Figure 3A). The observation of a mild glycosylation defect is supported by the changes within the N-glycan biosynthesis pathway seen on RNA level (Figure 3D). The notion of a secondary effect is supported by the observation of a normal Golgi membrane structure in V-8 fibroblasts (Figure 4A). We suspect that a permanent, though mild, impairment of glycoprotein biosynthesis might contribute to the clinical symptoms observed in the affected individual.

COPII consists of 3 principal components: SAR1, SEC23-SEC24, and SEC13-SEC31. SEC24 binds to SEC23 upon being recruited by active, GTP-bound SAR1 to the prebudding complex. The SEC13-SEC31 heteromer then interconnects the inner layer and forms the outer layer of the COPII coat. SAR1 self-assembly leads to the formation of a bottleneck and, ultimately, to vesicle fission (7). As expected, we observed absence of SEC24C from fibroblasts of individual V-8 (Figure 4B). The additional reduction of SEC23B and SEC13 in V-8 fibroblasts (Figure 4B) indicates that loss of 1 COPII component might destabilize the equilibrium of COPII-associated proteins in the cell, which might contribute to the observed delay in ER-Golgi trafficking (Figure 4, C and D). The mild delay in Golgi decompaction observed in the patient cells after treatment with BFA may either indicate an additional role for SEC24C in retrograde transport or be secondary to the deregulation of protein transport and degradation also observed in the transcriptome data, which indicated activated proteasomal degradation as well as a deregulation of autophagosome and lysosome formation (Figure 3, B–D, and Supplemental Data 3).

The reduction of SEC23B presents an intriguing explanation for the macrocytic anemia in individual V-8, since biallelic variants in one of the SEC24 binding partners (16), *SEC23B*, cause congenital dyserythropoietic anemia type II (anemia, congenital dyserythropoietic, type II; CDAN2, OMIM 224100). The clinical overlap between CDAN2 and the phenotype of individual V-8 is reflected not only in the presence of anemia and splenomegaly but also in the observation of anisopoikilocytosis in peripheral blood (Supplemental Figure 1).

It has been shown that each of the 4 known human SEC24 paralogs (SEC24A–D) selectively sorts cargo molecules into membrane vesicles by preferential recognition of different protein sorting signals (1, 6, 17, 18). This cargo specificity may underlie the different phenotypic outcomes of variants in each paralog. Although the paralogs SEC24C and SEC24D show an overlap in cargo recognition (19), it has been shown that sec24C can only compensate for sec24D at early stages of development in zebrafish (8, 9). With further tissue differentiation, each paralog becomes indispensable, also in terms of spatiotemporal expression (20, 21). Although *Sec24c*-knockout mice die at early embryonic stages (22), a conditional knockout of *Sec24c* in mouse neural progenitors causes apoptotic cell death of postmitotic neurons and results in microcephaly and perinatal lethality (10). The rescue of these phenotypes by ectopic expression of Sec24d (10) indicates that Sec24c is the main Sec24 isoform in adult mouse neurons, thus underpinning the idea that, in addition to cargo selectivity, tissue specificity contributes to the phenotypic differences in SEC24-associated disorders (9). The observation of a critical role of *Sec24c* in mouse brain development is in line with the observed phenotypes in our patient, especially the severe developmental delay, microcephaly, structural brain anomalies, and seizures.

To address potential functional redundancy, we have evaluated phenotypes of single *sec24C* and *sec24D* CRISPR/Cas9 knockout zebrafish models and double *sec24C sec24D* knockout animals. We observed differences in lens maturation, cerebellum morphology, and locomotion (Figure 5, A–J), both in the single-knockout animals and in the double mutants. The presence of these phenotypes in the single-knockout animals indicates that the loss of sec24C cannot be fully compensated for by other COPII components. Although a likely compensatory increase of SEC24D expression was observed on RNA and protein level in patient fibroblasts, the observed delay in ER-to-Golgi trafficking in sec24C-deficient fibroblasts (Figure 4, C and D), as well as the reduction of the cell membrane abundance of surface protein CD55 (Figure 4E), also argue for incomplete functional redundancy.

Congenital cataracts are a characteristic feature of the *SEC24C*-associated phenotype, which was present in all affected family members and reproduced in the *sec24C*-knockout zebrafish model (Figure 5, B and C). Cataracts are also a symptom of other, known phenotypes associated with COPII: *SEC23A*-associated craniolenticulosutural dysplasia (OMIM 607812) is characterized by cataracts, skeletal defects, and dysmorphism (23), and variants in *SEC31A* cause an autosomal-recessive neurodevelopmental disorder (OMIM 618651) that also includes cataracts as a prominent feature (24). As individuals harboring pathogenic variants in *TRAPPC11*, another component of the secretory pathway, also show congenital cataracts (20–22), we conclude that the secretory pathway in general and, more specifically, COPII-dependent secretion may play an important role in the embryonic development of the ocular lens.

Another prominent symptom observed in individual V-8 was low muscle tone. We explored this phenotype by testing larval movements in *sec24C*-deficient and double-knockout zebrafish larvae. Consistent with the human phenotype, *sec24C*-deficient zebrafish were almost immotile (Figure 5D). Normal muscle patterning, differentiation, and skeletal muscle volume (Figure 5, G and H, and Supplemental Figure 4D) argued against structural musculoskeletal changes as the cause of reduced zebrafish motility. Consistent with the human phenotype, we observed reduced cerebellar width in the *sec24C*-deficient and the double-mutant models, which, together with the brain malformations and the tongue fasciculations observed in individual V-8, points to neural deficits as the underlying cause of impaired motor function.

The complex interactions of the pathways and cellular components impacted by SEC24C loss of function need to be addressed in a follow-up study, exploring additional in vivo models.

Here, we present the human phenotype corresponding to a homozygous loss-of-function variant in the COPII complex member *SEC24C*. We describe a syndromic disorder characterized by congenital cataracts, dysmorphic features, severe developmental delay and intellectual disability, anemia, epilepsy, congenital microcephaly, hearing loss, and structural brain anomalies, and we explore the role of SEC24C in development and cell biology with regard to the reported symptoms. We show that loss of SEC24C causes trafficking and glycosylation defects, as well as alterations in cell surface proteins and deregulation of vital intracellular pathways in patient cells, and document that the human phenotype is partially reflected in the zebrafish model showing lens defects, cerebellar abnormalities, and motor dysfunction. This report adds another piece of knowledge to the puzzle that is the relationship between the secretory pathway and human disease and lays the cornerstone for further scientific investigations of *SEC24C*-related phenotypes.

## Methods

### Sex as a biological variable

Our study examined male and female human probands, and similar findings are reported for both sexes. For the animal studies, we used zebrafish larvae at very early developmental stages (4–5 dpf). Zebrafish sex differentiation is not completed at this stage. Therefore, sex was not considered as a biological variable for the zebrafish studies.

### Probands

We obtained written informed consent from all studied individuals or their legal representatives for the molecular genetic analyses, skin biopsy, and use of fibroblasts for research purposes and for publication of the results. We also obtained written informed consent for publication of photographs from the parents. Blood samples were collected from the studied individuals, and DNA was extracted from peripheral blood lymphocytes by standard extraction procedures.

### ES

Target enrichment was performed using the 96 rxnx Gen Exome Research Panel kit from Integrated DNA Technologies. ES was performed on the Illumina NovaSeq 6000. The raw data were processed by the varfeed pipeline (Limbus Medical Technologies GmbH) and reviewed and analyzed using the varvis (Limbus Medical Technologies GmbH) interface. Variant annotation for varvis was performed with the software allexes (Limbus Medical Technologies GmbH).

We filtered the identified variants against the databases of normal variation, such as dbSNP 135 and gnomAD (see section “Databases” below for URLs), as well as the allexes in-house database. Due to the consanguineous background, we used an inheritance-based approach, filtering for and prioritizing homozygous variants in individual V-8.

### Long-read genome sequencing

Whole-genome HiFi sequencing was performed on human high–molecular weight (HMW) DNA extracted from fibroblasts of individual V-8 using the SMRTbell preparation kit 3.0 (PacBio). In summary, 6 μg of HMW DNA was sheared with hydropores (Megaruptor 3 DNAFluid+ and the Megaruptor 3 shearing-kit from Diagenode) to 20 kb. The DNA damage and fragment ends were repaired, barcoded adapters were ligated, and fragments were cleaned. A nuclease treatment was used to remove incomplete SMRTbell templates. The required reagents were included in the SMRTbell prep kit 3.0, and barcodes were used from the SMRTbell adapter index plate 96A. Subsequently, large-insert SMRTbell libraries were achieved by using a size selection cutoff of 10 kb with the BluePippin system (SageScience). SMRT sequencing was performed with the Sequel II system on 3 SMRT Cells 8M (PacBio), using a 30-hour movie time, 2-hour immobilization time, and 2-hour pre-extension time with a targeted on-plate loading concentration of 85 pM. Circular consensus sequencing reads were generated, and demultiplexing was performed using standard settings in SMRTLink v11.0 (PacBio), with a minimum of 3 passes and minimum read quality of 0.99. Raw lrWGS data processing was carried out at the production site West German Genome Center; subsequent bioinformatics analyses and variant detection were performed at the Institute of Human Genetics Göttingen with an in-house pipeline for PacBio long-read sequencing data (PacBioinfAnalyzer.py), as described below. Variant interpretation was performed using the Geneyx Analysis software (Geneyx Genomex Ltd).

### In-house bioinformatic pipeline for PacBio sequencing data

Variant calling, phasing, and annotation were performed using the pipeline PacBioinfAnalyzer.py, specifically developed for PacBio sequencing data. The pipeline is written in Python, and all bioinformatics tools used for the pipeline are either containerized in Docker or available within specific open source Python environments.

PacBioinfAnalyzer.py emphasizes quality control using the NanoPlot Python library (25). For variant calling, the pipeline employs DeepVariant (26) for small variant detection and pbSV (27) for structural variant calling. Phasing analysis is performed using WhatsHap (28). Variant annotation is supported by a detailed analysis using svmatch (29) and a third party platform for next-generation sequencing data analysis and interpretation (Geneyx Genomex Ltd). Prior to data analysis, PacBioinfAnalyzer.py was evaluated using the Genome in a Bottle benchmark datasets for HG002. The tool achieved precision and specificity of 99.97% and 99.93% for SNVs and 95.05% and 88.94% for SVs, respectively.

### Real-time qPCR

We performed a real-time qPCR on RNA from fibroblasts of individual V-8 using the QuantStudio 5 Real-Time PCR System, 384-well (Thermo Fisher Scientific). Quantinova SYBR Green PCR Kit (QIAGEN) was used for the qPCR. The qPCR assay was based on amplification and quantification of *SEC24C* in relation to a reference gene, *ACTB*. The primers for *SEC24C* and *ACTB* were designed using Primer-BLAST (https://www.ncbi.nlm.nih.gov/tools/primer-blast/) and were purchased from Eurofins. Three pairs of the primers were used for the *SEC24C* amplification, SEC24C_c1F: GCAAACACCTCCCCAAGGTA, SEC24C_c1R: AAACTGGCCATATGCAGCCT, SEC24C_c2F: TTGAAGGAGGGAGGCGTTTC, SEC24C_c2R: TCAGGGCGGTCATAAGCATC, SEC24C_c3F: AAGCATGACGATCGGCTCAA, and SEC24C_c3R: GGCCAGATTATGGATGCGGA. The sequences of the primers for *ACTB* were ACTB_cDNA_qF: CTTCCAGCCTTCCTTCCTGG and ACTB_cDNA_qR: CTGTGTTGGCGTACAGGTCT. Each reaction had 10 mL volume containing 5 mL of Master Mix buffer and 5 pmol of the *SEC24C* and *ACTB* primers. cDNA was synthesized from 100 ng/μL of RNA, and 1 μL diluted cDNA (1/10) was used as template. Thermal cycling was performed for 2 minutes at 50°C, 10 minutes at 95°C, and then 40 cycles for 15 seconds at 95°C, for 15 seconds at 55°C, and for 15 seconds at 72°C. The threshold cycle parameter (Ct) was defined as the point at which the amplification plot — representing the fluorescence generated by dsDNA-binding SYBR Green as a function of the cycle number — passed a fixed threshold above baseline. Each replicate was normalized to *SEC24C* to obtain a ΔCt (*ACTB* Ct – *SEC24C* Ct).

### LC-MS analysis of whole serum N-glycans

LC-MS analysis of N-glycans derived from whole serum glycoproteins of a control pool (*n* = 100) and individual V-8 were conducted by the GlycoWorks RapiFluor-MS N-Glykan Kit (Waters) on an Integrated UPLC-FLR/QTOF mass spectrometry system with integrated software (BioAccord, Waters) as described previously (30).

### Transcriptome analysis

Total RNA samples were quantified using a Qubit Fluorometer, and RNA integrity was checked on a TapeStation (Agilent Technologies). Double-indexed stranded mRNA-Seq libraries were prepared using the Illumina Stranded mRNA Prep kit (50040534), starting from 1,000 ng of input material according to the manufacturer’s instructions. Libraries were equimolarly pooled based on Qubit concentration measurements and TapeStation size distributions. The loading concentration of the pool was determined using a qPCR assay (Roche, 7960573001). Libraries were then sequenced on the Illumina NovaSeq X Plus platform using PE100 sequencing mode, with a target of 50 million reads per library.

Bioinformatic analyses were performed using the R statistical software (v4.3.2; R Core Team) (31). Paired-end, 101–base pair reads were mapped to the Ensembl human reference genome GRCh38.104 using STAR (v2.7.9a) (32). Transcript quantification was carried out with RSEM (v1.2.31) (33), and differential expression analysis was conducted using the edgeR package (v3.8.6) (34). *P* values were adjusted for multiple testing using the Benjamini-Hochberg method, and a false discovery rate threshold of 5% was used to identify significant results. Significant gene sets were selected for KEGG-based enrichment analysis, which was performed using the clusterProfiler package (v4.8.3) (35). KEGG terms with an adjusted *P* < 0.05 (Benjamini-Hochberg) were considered significantly enriched. Heatmaps were generated using correlation distance and complete linkage.

### Western blot analysis

Samples from control and V-8 fibroblasts (30 μg total protein) were analyzed on either 8% or 15% SDS-PAGE. The proteins were transferred to a PVDF membrane (MilliporeSigma) for 1 hour at 100 V. Membranes were then blocked with 5% skim milk powder in PBS with 0.1% Tween 20 (vol/vol) (PBS-T) for 1 hour at room temperature. The list of the primary and secondary antibodies used and their dilutions is shown in Supplemental Table 2. All primary antibodies were incubated in PBS-T overnight at 4°C, except for anti-tubulin antibody, which was incubated for 30 minutes at room temperature. The secondary antibodies were incubated for 45 minutes at room temperature. Membranes were then incubated with ECL reagent (GE Healthcare, now Cytiva) and detected using an Amersham Imager 600.

### RUSH assay

The RUSH assay was performed as previously described (36). Control and V-8 fibroblasts were transfected by electroporation with Str-KDEL_ST-SBP-eGFP plasmid (Addgene plasmid 65264). The plasmid coexpresses a Golgi-localized enzyme (sialyl transferase-eGFP fused to SBPs) as well as KDEL-tagged streptavidin, necessary for the ER retention of ST-eGFP. Simultaneous release of the reporter from the ER was achieved by the addition of 40 μM biotin, and live cells were monitored by fluorescence microscopy every 2 minutes for 60 minutes. Images were obtained on a Nikon Livescan sweptfield confocal microscope with a 40× objective lens (NA 0.95). The quantifications and analysis were performed as described before (37). The kinetics of ST-eGFP trafficking represents a change in the ratio over time (0 to 30 minutes).

### Immunofluorescence microscopy

Fibroblasts were grown on coverslips for 36–48 hours. The cells were then washed with PBS and fixed with 4% paraformaldehyde (PFA) for 15 minutes at room temperature. Following quenching with 0.1 M glycine for 10 minutes and permeabilization with 0.1% Triton X-100 for 7 minutes, the fibroblasts were blocked in 5% normal goat serum (Cell Signaling Technology) for 40 minutes at room temperature. The primary antibodies (mouse anti-p115 and rabbit anti-ManII, see Supplemental Table 2) were diluted in 5% normal goat serum, added to coverslips, and incubated overnight at 4°C. Cells were then washed twice with PBS for a total of 10 minutes and incubated for 40 minutes at room temperature with the secondary antibodies. Cells were then washed 3 times for 10 minutes, and Hoechst 33342 was added during the first wash at a final concentration of 0.1 μg/mL. The coverslips were then dried and mounted with ProLong Gold Antifade reagent (Life Technologies). All 16-bit images (1,024 × 1,024 pixel resolution) were recorded on a Nikon C2 laser scanning confocal microscope fitted with a 63× Plan Apo I, NA1.4 objective (Nikon) controlled by NIS-Elements C 4.4 software. The images were acquired with a 0.2 μm increment size.

### GPI-anchored proteins

To analyze the expression of GPI-anchored proteins, expression levels of 3 surface antigens including CD55, CD73, and CD59 were analyzed. Briefly, cells were cultured in complete DMEM, supplemented with 10% FBS in T75 flasks. Upon attaining 90% confluence, cells were harvested using trypsin-EDTA (0.05%), centrifuged at 500*g* for 3.5 minutes, and washed twice with PBS and 1 time with FACS buffer (2 mM HEPES, 2 mM EDTA, 2% FBS in PBS). To obtain a uniform single-cell suspension, cells were filtered through a Corning 40 μm Cell Strainer and cell counting was performed. Aliquots of 100,000 cells in triplicates were prepared in a total volume of 100 μL of FACS buffer. The cells were either unstained or stained with fluorophore-conjugated antibodies (CD55-PE [BD 56190], CD59-FITC [BD 560954], CD73-PE [BD 561014]) or with the same concentration of corresponding isotype controls for 30 minutes in the dark at room temperature. Following staining, the cells were washed twice and resuspended in 1 mL of FACS buffer. Flow cytometric analysis was conducted using the Attune CytPix Flow Cytometer (Invitrogen). After applying appropriate gating, 20,000 events were recorded at a flow rate of 200 μL/min. MFI of the isotype control was subtracted from the MFI of the corresponding sample, and absolute MFI of 3 replicates ± SD was reported.

### BFA assay

Fibroblasts grown on coverslips in 6-well plates were treated with 5 μg/mL of BFA (Bioshop, BRE222) for 15 minutes. Samples were fixed after 0, 3, 6, 12, and 15 minutes of incubation with BFA with 4% PFA for 20 minutes until subsequent immunostaining. For BFA withdrawal, cells were incubated with BFA for 15 minutes, were washed 2 times with warm PBS, and were maintained in complete DMEM supplemented with 10% FBS. At different time intervals (every 15–20 minutes), cells were fixed and were washed 2 times with PBS. After fixation, cells were treated with 0.1 M glycine for 10 minutes, permeabilized with 0.1% Triton X-100 for 8 minutes, and blocked with 5% normal goat serum for 40 minutes at room temperature. Staining was performed using anti-ManII with a dilution of 1:200 in 5% normal goat serum in PBS for 2 hours, and secondary antibody was anti-rabbit Alexa Fluor 647 (Invitrogen, A21245) with a dilution of 1:200 for a duration of 40 minutes. After subsequent washing, coverslips were mounted using ProLong Gold Antifade and visualized by confocal microscopy.

### Confocal microscopy

Images were obtained using an Olympus FV10i confocal laser scanning microscope. High-resolution 1,024 × 1,024 3D images with 10 *z*-stacks of 0.2 μm optical sections were captured using a 60× objective (NA 1.35) with balanced scan speed. The acquired images were processed using ImageJ (NIH). Each *z*-stack was merged using maximum-intensity projection. For each sample at least 6 fields of view (35–60 cells) were analyzed in duplicates using a macro script. Briefly, individual cells were outlined, and an appropriate thresholding (Huang dark) was applied to mask the Golgi area. Automatic particle counting was performed in segmented images to measure Golgi compaction index (calculated as roundness = 4π × area/perimeter^2^).

### Databases

The following databases were used for this study: DECIPHER: https://www.deciphergenomics.org; dbSNP: https://www.ncbi.nlm.nih.gov/snp/; Ensembl: https://www.ensembl.org/index.html; gnomAD: https://gnomad.broadinstitute.org/; Human Gene Mutation Database: https://www.hgmd.cf.ac.uk/ac/index.php; UCSC Genome Browser: http://genome.ucsc.edu/; UniProt: https://www.uniprot.org/; and PubMed: http://www.ncbi.nlm.nih.gov/pubmed/

### Protein motif search

Information on SEC24C protein domains, as displayed in Figure 3A, and putative binding motifs were collected from peer-reviewed publications (1, 38, 39), protein databases (https://www.uniprot.org/; http://www.hprd.org/), and a pfam-based protein domain search (https://www.genome.jp/tools/motif/; http://pfam.xfam.org/protein/P53992).

### Zebrafish studies

#### Fish maintenance and breeding.

Zebrafish (*Danio rerio*) were used in accordance with Vanderbilt University Institutional Animal Care and Use Committee (IACUC) guidelines and with an approval protocol. Zebrafish were raised under standard laboratory conditions at 28.5°C with a constant photoperiod (14 hours light/10 hours dark) as previously described (12). Larvae were staged at days postfertilization (dpf). Genotypes of the animals were determined by PCR and sequencing or phenotypic analysis.

The *sec24D*(–) line used in this study was the *N*-ethyl-*N*-nitrosourea–induced *bul^m421^* line as described previously (8). *Sec24D*(–) models were screened by phenotype. *Sec24C*(–) *sec24D*(–) double-deficient models were generated by injecting *sec24C* sgRNA into 1-cell–stage embryos generated from crosses of *bul^m421^* heterozygotes.

#### CRISPR/Cas9 genome editing.

CRISPR/Cas9 target sites in the zebrafish *sec24C* gene were identified using the CHOPCHOP web tool (26). sgRNA templates were designed as previously described (27). gRNAs were synthesized with the MEGAshortscript T7 transcription kit (AM1354, Thermo Fisher Scientific). One-cell–stage wild-type embryos were injected with 500 pg of purified Cas9 protein (CP01, PNA Bio Inc.) and 300 pg sgRNA. Efficiency of genome editing was assessed by heteroduplex assay (28) and direct Sanger sequencing of the targeted region.

We included Cas9-only and gRNA-only injections in the experimental design to control for potential nonspecific phenotypes. Injected embryos were grown to 4 or 5 dpf for phenotypic analysis.

Primers for genome editing were sgRNA a: 5′-GGGGGTGGCCATGTGCCATCGGG-3′ and sgRNA b: 5′-GGATCACACAGGGAAGAGAGTGG-3′; PCR primers were sgRNA a forward primer: 5′- GAGAATGAATGTCAACCAGCAA-3′; sgRNA a reverse primer: 5′-AACCCATCATAAACGTGTCTCC-3′; sgRNA b forward primer: 5′-ACGTTATGGTTTCTGGTCTGCT-3′; and sgRNA b reverse primer: 5′-ATTCAAATAAGCAATCCCATGC-3′.

#### Lens microscopy and image processing.

Injected zebrafish were grown to 4 dpf in embryo medium supplemented with 1-phenyl 2-thiourea, then were anesthetized with 0.15 mg/mL Tricaine (TRS1, Pentair) and mounted in 3% methylcellulose on a bridge slide for imaging. Images were acquired with ZEISS upright microscope (Imager) under bright-field illumination. Differential interference contrast was performed with both Plan Apo 20×/0.80 NA objective lens (ZEISS) and Flash 4 charge-coupled device camera (Hamamatsu). Images were acquired and formatted by Zen software (ZEISS). Lens artifacts were counted in digital images (1 eye per larvae) and analyzed by ImageJ-FIJI software package, as previously described (40). Data were analyzed using 2-tailed Student’s *t* test.

#### Locomotor behavior analysis.

Wild-type, *sec24C*(–), *sec24C*(–) *sec24D*(–), and *sec24D*(–) zebrafish larvae at the 5 dpf stage were placed in individual wells of a 48-well plate filled with embryo medium. Locomotor behavior of the larvae was induced by a single poke using a metal needle, and movement was recorded for 30 seconds, at 0.03 seconds per frame with an HRc camera mounted on Stemi-2000C stereoscope (ZEISS), at 0.65× magnification. Video recording was performed using ZEN2 software (Blue edition) with 1 × 1 binning, 1 ms exposure, B/W setting, at 1,388 × 1,040 pixel resolution. Movies were opened in ImageJ and analyzed with an MTrackJ plug-in. The position of each larva was manually tracked frame by frame. Total distance traveled by the larvae was obtained with MTrackJ’s Measure tool. Distance to previous position (D2P) was also recorded by MTrackJ for each single displacement event. Maximum D2P value (MaxD2P) of each larva was used to calculate maximum velocity reached during the travel by the following formula: MaxD2P/time between frames (0.03 seconds). A total of 48 videos were generated, accounting for 12 larvae in each experimental group. Initial (*x*, *y*) coordinates (generated by MTrackJ, Measure Tool, Points sheet) of each larva were taken as (0, 0) origin point, and all following coordinates were adjusted with reference to the origin, creating relative coordinates values, as previously described (16). Relative (*x*, *y*) coordinates were plotted in R using ggplot2 to display individual tracks. Upon completion of the analyses, 1 animal with highest and 1 animal with lowest values were removed from each experimental group to account for potential outliers and data from the remaining 10 animals per group are presented. Statistical analysis was done using the Mann-Whitney *U* test with a Bonferroni-corrected significance threshold (*P* < 0.0167) accounting for multiple tests.

#### Whole-mount immunofluorescence.

Zebrafish larvae at 5 dpf were fixed in Prefer Fixative (Anatech) for 48 hours, rinsed in PBS-0.1% Tween 20, postfixed in 4% PFA for 10 minutes, and then permeabilized in 0.5% Triton X-100 in PBS. Next, larvae were bleached with 1.5% H_2_O_2_/1% KOH, then rinsed, blocked in 10% goat serum, 1% DMSO, and 1% BSA and incubated in primary antibody (1:250 dilution) overnight at 4°C. Secondary antibody incubation (1:300 dilution) with fluorophore-conjugated antibodies was followed at 4°C overnight. After rinsing in PBS-0.1% Tween 20, DAPI staining was performed (1:5,000 dilution), and then larvae were postfixed in 4% PFA for 20 minutes, and subsequently, cleared and stored in 90% glycerol. Stained larvae were imaged on a Nikon Spinning Disk Confocal Microscope. Images are presented as maximum-intensity *z*-projections generated by Nikon NIS-Elements software. Primary antibodies used were: anti-myosin (Developmental Studies Hybridoma Bank MF20, AB_2147781) and anti–acetylated tubulin (MilliporeSigma T7451). Secondary antibodies used were anti-Mouse–Alexa Fluor 488 (Thermo Fisher Scientific A-21141) and anti-Mouse–Alexa Fluor 555 (Thermo Fisher Scientific A-21147). Alexa Fluor 488–conjugated WGA (Thermo Fisher Scientific W11261) incubation was performed at the secondary antibody incubation step with 1:300 dilution in blocking solution. DAPI counterstaining was performed after the secondary antibody step, by incubating for 20 minutes in 1:5,000 diluted DAPI in PBS-0.1% Tween 20.

#### Craniofacial muscle area measurement.

Whole-mount zebrafish larvae stained with MF20 were imaged on a Nikon Spinning Disk Confocal Microscope with Plan Apo Lambda ×10/0.45 NA objective. Maximum-intensity *z*-projections (100–200 μm depth) were created in Nikon NIS-Elements software and analyzed in ImageJ using freehand selection and measurement tools to measure the intermandibularis anterior (ima), intermadibularis posterior (imp), interhyoideus (ih), hyohyoideus (hh), and sternohyoideus (sh) muscles, as previously described (41). Statistical analysis was done using the 2-tailed Student’s *t* test with a Bonferroni-corrected significance threshold (*P* < 0.0125).

#### Cerebellum width.

Whole-mount zebrafish larvae stained with anti-acetylated tubulin were imaged on a Nikon Spinning Disk Confocal Microscope with Plan Apo Lambda 10×/0.45 N. A. and 20×/0.75 NA objectives. Maximum-intensity *z*-projections (~100 μm depth) were created in Nikon NIS-Elements software and analyzed in ImageJ using line measurement tools. Measurements were confirmed using samples stained with WGA. Statistical analysis was done using the 2-tailed Student’s *t* test with a Bonferroni-corrected significance threshold (*P* < 0.0125).

### Sources of animals and cell lines

Fibroblast cell lines of controls and of individual V-8 originate from the Institute of Human Genetics Göttingen. The zebrafish originated from and were maintained at Vanderbilt University Medical Center Aquatic Facility.

### Statistics

All statistical analyses were performed with GraphPad Prism 9.3.1. Lens artifact count, craniofacial muscle area, and cerebellum width were assessed by 2-tailed Student’s *t* test. Total distance traveled and maximum velocity were assessed by the 2-tailed Mann-Whitney *U* test. Significance thresholds were adjusted using Bonferroni correction for multiple comparisons when appropriate. *P* < 0.05 was considered statistically significant.

### Study approval

The study on the human individuals was performed according to the Declaration of Helsinki protocol and approved by the institutional ethics board at the University Medical Center Göttingen (Vote ref 3/2/16). Written informed consent was received for the use of the photographs, and the record of informed consent has been retained. The animal study was reviewed and approved by IACUC at Vanderbilt University Medical Center.

### Data availability

The *SEC24C* variant is available at the open access database ClinVar (https://www.ncbi.nlm.nih.gov/clinvar/) with accession ID SCV005627764.

The graphical abstract was created in BioRender, with an academic license, and is available at https://BioRender.com/w25t630

Experimental data are available in the Supporting Data Values XLS file.

Next-generation sequencing data of the human participant are retained at the study institutions and can be made available from the corresponding author upon request. The data are not publicly available due to privacy restrictions.

More details concerning the pipeline PacBioinfAnalyzer.py can be made available from the corresponding author upon request.

## Author contributions

NB coordinated the study; analyzed data; wrote the manuscript, with contributions from all authors; and designed the graphical abstract. BC gathered clinical information and patient samples. THN performed zebrafish experiments. MPM, MM, and DSD performed in vitro studies. AW performed bioinformatic analyses. OGG and LC performed in vitro studies. JS assisted with data analysis and phenotyping. DC supervised and performed zebrafish experiments. KK, TEW, and DW performed and/or supervised long-read genome sequencing. JA and TB performed the transcriptome analysis. AZ codeveloped the in-house PacBio long-read analysis pipeline together with AW. YL analyzed the exome data and performed qPCR. DK and GH were involved in clinical management and phenotyping. CT performed and interpreted LC-MS. MS planned and supervised Western blot analyses, immunostainings, and RUSH assay. EWK designed and supervised zebrafish experiments and contributed resources. GY performed data analysis. BW designed and supervised the study.

## Supplementary Material

Supplemental data

Supplemental data set 1

Supplemental data set 2

Supplemental data set 3

Unedited blot and gel images

Supporting data values

## Version 1 03/25/2025

In-Press Preview

## Version 2 05/08/2025

Electronic publication
