# Supplementary material for: SEC24C deficiency causes trafficking and glycosylation abnormalities in an epileptic encephalopathy with cataracts and dyserythropoeisis
Source: JCI Insight. 2025 Mar 25;10(9):e173484. doi: 10.1172/jci.insight.173484 (PMC12128993; doi:10.1172/jci.insight.173484)
Supplement: Supplemental data [file jciinsight-10-173484-s115.pdf]

Supplemental Table 1. Laboratory results of individual V-8.

Value	32 months	18 months	Previous results	3 months	Reference value	Unit
**ALP**		586			(104-345)	U/L
**GGT**		42			(3-22)	U/L
**Bilirubin total**		0.85			(0.3-1.2)	mg/dL
**Direct bil.**		0.152 mg/dL			(0-0.2)	mg/dL
**Indirect bil.**		0.7 mg/dL			(0-1.2)	mg/dL
**AFP**				8726	(0-9)	ng/ml
**Ferritin**				2800	(11-53)	µg/l
**Hb**	11.9	10.8	9.0/10.1/10.1		(11.1-14.1)	gr/dL
**Htc.**	36.1	32.9	27.3 /29.9 /29.0		(30-38)	%
**MCV**	101.2	97.3	85.4/88.6/ 88.5		(72-84)	fL
**MCH**	33.3	32.0	26.3/30.0/30.0		(25-29)	pg
**MCHC**	32.9	33.0	33.1/3.7/34.0		(32-36)	g/dL
**RDW**	18.6	17.7	16.1/16.7/17.0		(11.8-14.3)	%
**Trombocytes**	147	241			(200-460)	$*10^9$ / L
**MPV**	13.9				(7.2-11.2)	fL
**Lymphocytes**	24.8				(28-59)	%

Supplemental Table 2. Antibodies.

Antigen	Type	Host	Dilution	Observed MW (kDa)	Catalog number	Source
**ManII**	P	r	1 : 200	N/A	N/A	Moreman K
**membrin**	M	m	1 : 200	N/A	Ab13511	Abcam
**Sar1B**	P	r	1 : 1000	29kDa	C16030	Assay BioTech
**Sec13**	M	m	1 : 500	38kDa	sc514308	Santa Cruz
**Sec23B**	P	r	1 : 1000	80kDa	N/A	Schekman R
**Sec24C**	P	r	1 : 1000	118kDa	A10797	Abclonal
**Sec24D**	P	r	1:1000	115 kDa	A21139	Abclonal
**Sec31A**	M	m	1 : 500	135kDa	sc-376587	Santa Cruz
**tubulin**	M	m	1 : 10000	50kDa	T6199	Sigma-Aldrich
**HRP-labeled anti-mouse**	HCA	g	1 : 5000	N/A	KP-474-1806	KPL
**HRP-labeled anti-rabbit**	HCA	g	1 : 5000	N/A	KP-474-1506	KPL
**Alexa Fluor 488 anti–mouse**	HCA	g	1 : 500	N/A	A-11031	Life Technologies
**Alexa Fluor 647 anti–rabbit**	HCA	g	1 : 500	N/A	A-21235	Life Technologies
**PE Mouse Anti-Human CD73**	M	m	1.25ng/µL	N/A	561014	BD Pharmingen™
**PE Mouse Anti-Human CD55**	M	m	2.5ng/µL	N/A	561901	BD Pharmingen™
**FITC Mouse Anti-Human CD59**	M	m	5ng/µL	N/A	560954	BD Pharmingen™
**PE Mouse IgG2a ĸ isotope control**	N/A	m	2.5ng/µL	N/A	Lot: 331124 Part: 26.1055	Sony Biotechnology
**PE Mouse IgG1 ĸ isotope control**	N/A	m	1.25ng/µL	N/A	55749	BD Pharmingen™
**FITC Mouse IgG2a ĸ isotope control**	N/A	m	5ng/µL	N/A	Lot: 290553, Part: 2601035	Sony Biotechnology

Supplemental Figure 1. Peripheral blood smear of individual V-8.

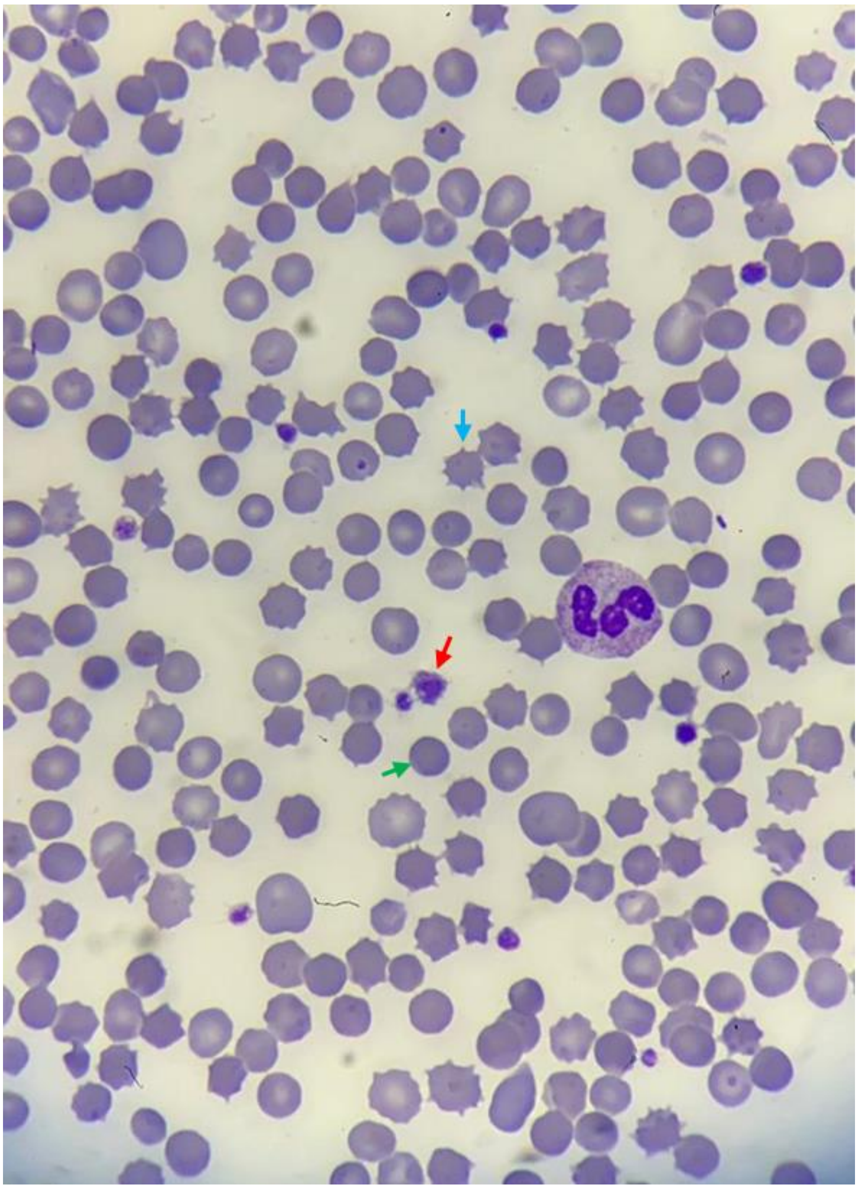

Supplemental Figure 1. Peripheral blood smear. Note spherocytosis (exemplary cell marked with green arrow), anisocytosis, spheroacanthocytes (exemplary cell marked with blue arrow), and large platelets (exemplary cell marked with red arrow).

Supplemental Figure 2. qPCR results for *SEC24C* mRNA.

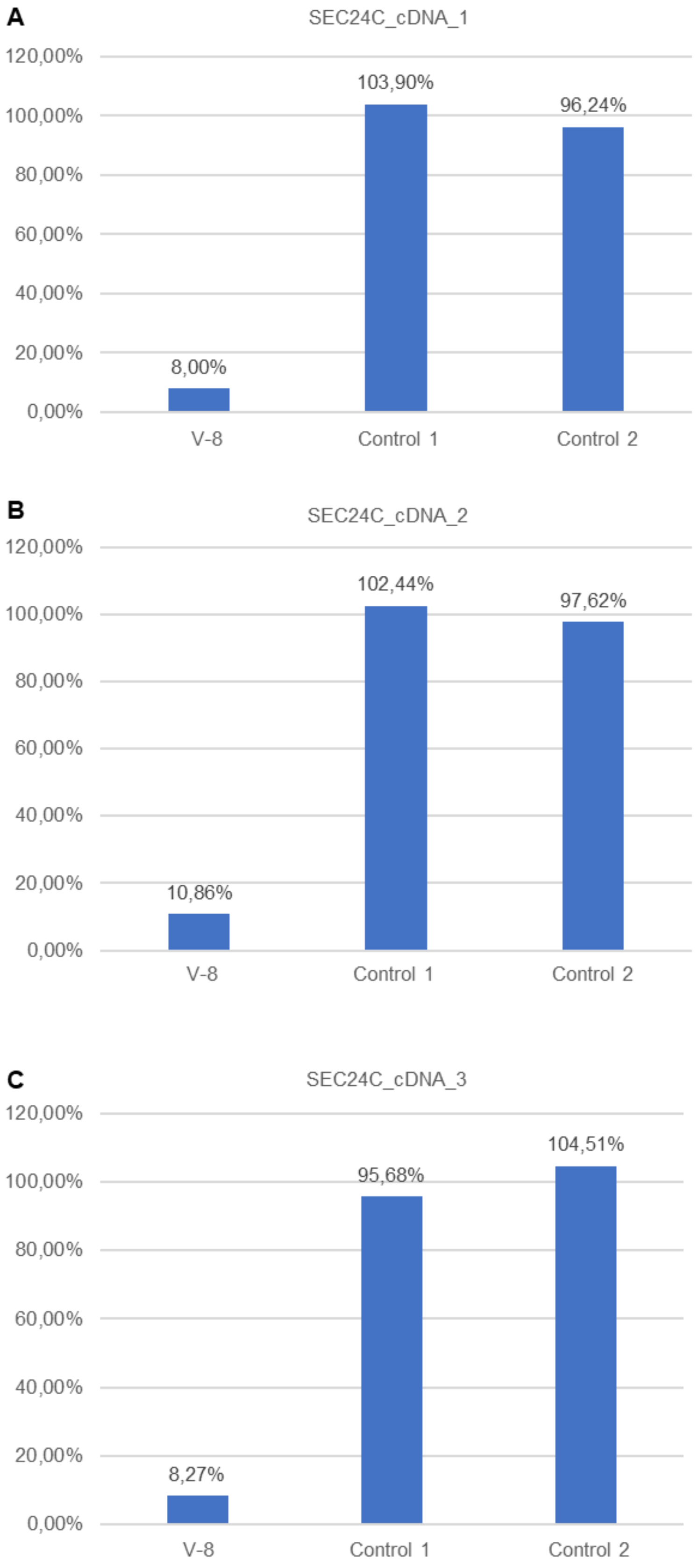

Supplemental Figure 2. qPCR results. (A-C) qPCR analysis from three independent experiments with different primer pairs showed reduced mRNA levels between 8% and 10.86%.

Supplemental Figure 3. BFA treatment of SEC24C deficient fibroblasts.

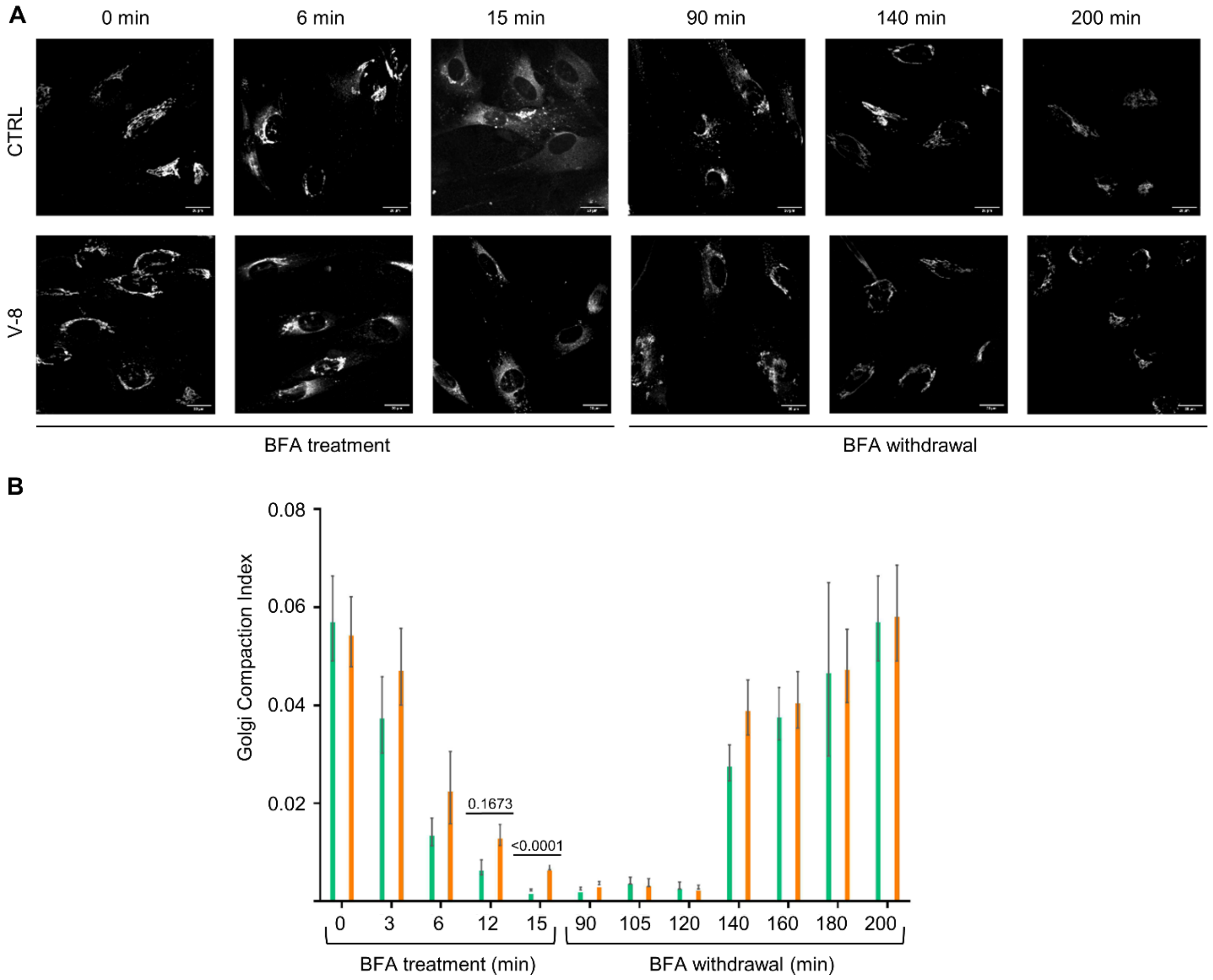

Supplemental Figure 3. BFA experiment. (A) Human fibroblasts from control and V-8 were treated with brefeldin A (BFA) for 15 minutes, BFA was washed out and the cells were then maintained in BFA-free DMEM. Samples were fixed at different time points during both treatment and withdrawal. Golgi was stained using Mannosidase II antibody to monitor the state of the Golgi during disassembly (BFA treatment) and reassembly (BFA withdrawal). Confocal microscopy was used to obtain images. The scale bar is 20μm. (B) The compaction of Golgi was quantitated using the circularity formula (4π × Area/Perimeter2) using ImageJ software. Quantitation was performed for 35-50 cells and data is presented as mean ± SEM. Green bars are control and orange bars are V-8. Bonferroni's multiple comparisons test was used to show the data significance level.

Supplemental Figure 4. Additional results from zebrafish studies.

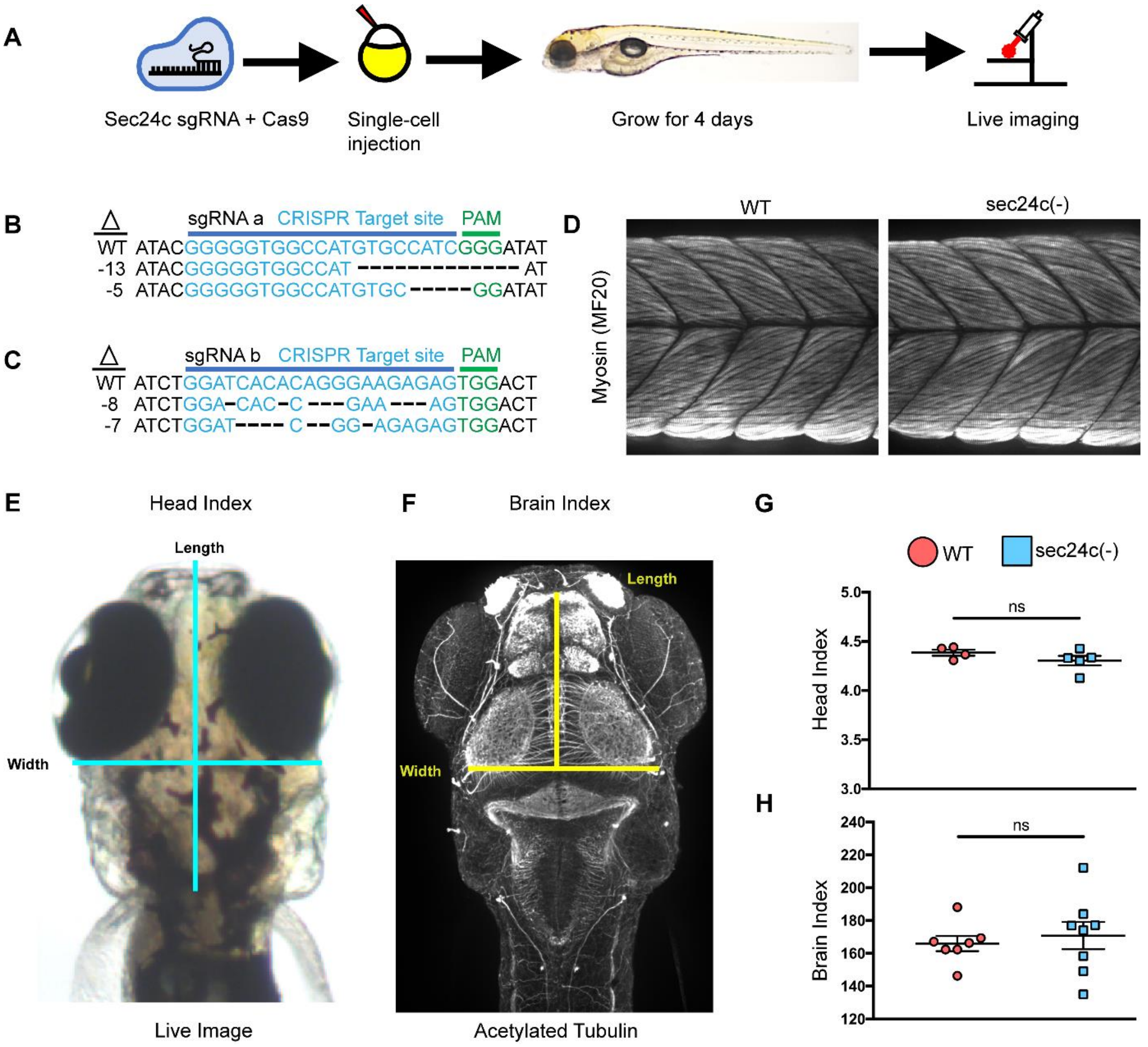

Supplemental Figure 4. Additional results from zebrafish studies. Sec24C edited zebrafish show normal trunk muscle development, and head and brain indices. (A) Strategy for generation of transient CRISPR models through single-cell injections of single guide RNA (sgRNA a and b) by CAS9 editing. (B) Representative mutations generated by sgRNA a, and (C) sgRNA b after sequencing genomic DNA isolated from individual embryos. All phenotypically altered embryos carried edited sequences with most changes resulting in small deletions in the PAM targeted region. (D) Whole mount fluorescence staining for muscle myosin (MF20 antibody) at 5 dpf (lateral view) shows no differences in trunk muscle development between WT and *sec24C*-edited larvae. (E) Head index was used to standardize larvae head. The length was measured from tip of jaw to base of fins, and width was defined as the distance between ear regions right behind eyes; here marked with blue lines on the image of wild type larvae. (F) Brain index used to describe size of forebrain and midbrain of zebrafish larvae was defined as length from the most anterior forebrain (vertical line) to base of optic tectum, and width as the distance at the base of optic tecta. Measurements were made using 5 dpf images of acetylated tubulin stained larvae. (G) Head index graph of WT (n=4) and transient *sec24C*(-) zebrafish larvae (n=5). No significant difference was detected between groups by student's T test. (H) Brain index graph of WT (n=7) and transient *sec24C*(-) zebrafish larvae (n=8). No significant difference was detected by student's T test.

Supplemental Methods pertaining to Supplemental Figure 4 D-F

(D) Whole mount zebrafish larvae stained with MF20 were imaged on a Nikon Spinning Disk Confocal Microscope with Plan Apo Lambda 4x objective. Maximum intensity projections Z-stack (100 µm - 200 µm depth) were created in Nikon Elements software and analyzed in ImageJ (NIH). (E) Live zebrafish larvae at 5 dpf were anesthetized in Tricaine (MS-222) solution and positioned in methylcellulose for imaging. Pictures were taken with an HRc camera mounted on Stemi-2000C stereoscope (Zeiss) at 1.25X magnification. Measurements were taken using ImageJ line measurement tool. Ratio of length to width was graphed and statistically analyzed by student's T test. (F) Whole mount zebrafish larvae stained with acetylated tubulin were imaged on a Nikon Spinning Disk Confocal Microscope with Plan Apo Lambda 10x/0.45 N.A. objective. Maximum intensity projection Z-stack (~100 µm depth) were created in Nikon Elements software and analyzed in ImageJ using line measurement tools. Ratio of length to width was graphed and statistically analyzed by student's T test.
