# Supplementary figures and images for: SEC24C deficiency causes trafficking and glycosylation abnormalities in an epileptic encephalopathy with cataracts and dyserythropoeisis
Source: JCI Insight. 2025 Mar 25;10(9):e173484. doi: 10.1172/jci.insight.173484 (PMC12128993; doi:10.1172/jci.insight.173484)

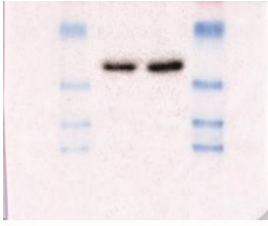

SEC24D

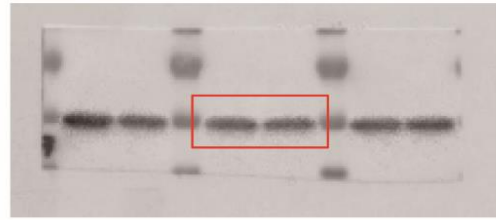

tubulin

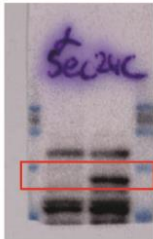

SEC24C

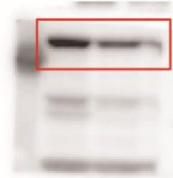

SEC23B

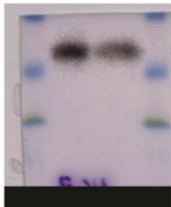

SEC13

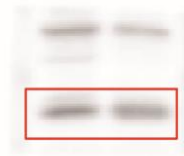

SAR1

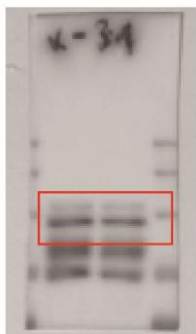

SEC31A

Supplement: Unedited blot and gel images [file jciinsight-10-173484-s119.pdf]
